# Modified Dachengqi Decoction ameliorates sepsis-induced lung injury via the gut microbiota-bile acid axis

**DOI:** 10.3389/fcimb.2026.1661639

**Published:** 2026-02-06

**Authors:** Lulu Wu, Weihang Peng, Ya Li, Liyuan Yu, Peiying Huang, Ye Ye, Yuchao Feng, Bojun Chen, Li Chen

**Affiliations:** 1 The Second Clinical School of Medicine, Guangzhou University of Chinese Medicine, Guangzhou, China; 2 Guangdong Provincial Academy of Traditional Chinese Medicine, Clinical Research Team of Prevention and Treatment of Cardiac Emergencies with Traditional Chinese Medicine, Guangzhou, China; 3 Emergency Department, Guangdong Provincial Hospital of Traditional Chinese Medicine, Guangzhou, China

**Keywords:** bile acid metabolism, FXR/TLR4/MYD88, gut microbiota, Modified Dachengqi Decoction, neutrophil extracellular traps, sepsis-induced acute lung injury

## Abstract

**Background:**

Sepsis-induced acute lung injury (SI-ALI) is associated with high mortality. The gut microbiota-bile acid axis plays a critical role in regulating host inflammatory responses; however, the mechanism of action of traditional Chinese medicine (TCM) compounds targeting this axis remains unclear.

**Aim:**

This study aimed to systematically evaluate the protective effects of Modified DaChengqi Decoction (MDD) against lipopolysaccharide (LPS)-induced SI-ALI and to elucidate its underlying mechanism in modulating inflammation and neutrophil extracellular traps (NETs) through the regulation of gut microbiota and bile acid metabolism.

**Methods:**

An LPS-induced mouse model of SI-ALI was established. Mice were orally administered MDD, and 72−h survival rate, lung function, histopathology, and inflammatory cytokine levels were assessed. Fecal 16S rRNA sequencing and targeted bile acid metabolomics were combined to analyze changes in the microbiota and metabolites. Network pharmacology was employed to screen key targets, followed by experimental validation using Western blotting, immunohistochemistry, and ELISA to confirm candidate pathways.

**Results:**

Compared with the model group, MDD significantly improved survival and lung function, alleviated pulmonary inflammation and vascular permeability. Microbiomic analysis revealed that MDD downregulated the abundance of *Parabacteroides* and *Bacteroides*. Targeted metabolomics showed that MDD markedly altered the levels of several primary and secondary bile acids, mainly including glycoursodeoxycholic acid (GUDCA), taurochenodesoxycholic acid (TCDCA), chenodeoxycholic acid (CDCA), and taurocholic acid (TCA). Molecular validation demonstrated that the nuclear receptor FXR was significantly upregulated, while the TLR4 and downstream MYD88-NF−κB/JNK signaling pathways were inhibited. Additionally, the expression of PAD4 and CitH3 as well as NETs formation were reduced.

**Conclusion:**

MDD can alleviate LPS-induced SI-ALI by modulating the gut microbiota-bile acid metabolism, activating FXR, and thereby suppressing the TLR4/MYD88−mediated inflammatory cascade and NETs generation.

## 1 Introduction

Sepsis is a severe condition that poses a significant threat to global health, with persistently high incidence and mortality rates. The excessive inflammatory response triggered by sepsis can lead to multiple organ failure (Singer et al., 2016). Notably, the lungs are among the organs most susceptible to septic injury, ultimately resulting in sepsis-induced acute lung injury(SI-ALI). Approximately 45% of sepsis patients progress to acute respiratory distress syndrome (ARDS), characterized by disruption of the pulmonary endothelial barrier and diffuse lung injury (Li N, et al., 2023). Despite decades of research on SI-ALI, outstanding breakthroughs in clinical treatment remain lacking, highlighting an urgent need to develop novel pharmaceuticals and innovative therapeutic strategies.

Recent studies have increasingly emphasized the concept of “gut-derived sepsis”, underlining the critical role of the gut in driving multi-organ dysfunction during sepsis through complex organ crosstalk mechanisms (Li D, et al., 2024).Gut dysbiosis is a pivotal contributor to sepsis progression by promoting pathogenic bacterial proliferation, triggering excessive pro-inflammatory immune responses, and reducing the production of beneficial metabolites, thereby increasing host susceptibility (Charitos et al., 2025). This dysbiosis also profoundly disrupts host bile acid metabolic homeostasis. Dysregulation of bile acid metabolism plays a crucial role in sepsis-induced lung injury. Recent reviews emphasize the central role of bile acids and their receptors, especially FXR and TGR5, in coordinating metabolic homeostasis and inflammatory responses, and highlight that modulation of the bile acid-FXR signaling axis is a promising therapeutic strategy to suppress systemic inflammation and treat metabolic disorders (Fleishman and Kumar, 2024; Li et al., 2024).Mechanistically, bile acids exert broad regulatory effects on host metabolism, immunity, and inflammation through activation of nuclear receptors such as FXR and PXR as well as the G protein–coupled receptor TGR5 (Thomas, 2017; Li and Chiang, 2024). Sepsis itself exacerbates dysbiosis and bile acid imbalance (Haak and Wiersinga, 2017), forming a vicious cycle of dysbiosis-metabolic disorder-sepsis aggravation. The cohort observations and animal experiments have further confirmed that following the onset of sepsis, the composition of the gut microbiota and the profile of bile acids undergo rapid alterations. These changes are closely associated with elevated systemic inflammatory markers and adverse clinical outcomes. This suggests a potential causal pathway in which infection may induce dysbiosis, subsequently leading to disturbances in bile acid metabolism, and ultimately exacerbating the systemic inflammatory response (Li Z. et al., 2024; Shang et al., 2025). Therapeutic strategies targeting gut microbiota and bile acid metabolism modulation urgently require further exploration. In this context, traditional Chinese medicine (TCM), with its long-standing history, has gradually demonstrated unique advantages in the clinical treatment of sepsis. TCM has been observed to induce corresponding changes in gut microbiota while ameliorating organ damage caused by sepsis (Fan et al., 2023), exerting its effects through multiple mechanisms, encompassing the regulation of immune responses and anti-inflammatory effects. Although the significance of gut microbiota and their metabolites in sepsis and multiple organ dysfunction has been revealed by previous studies, the mechanism by which gut microbiota−associated bile acid imbalance disrupts vascular endothelial structure in distant organs through interactions between nuclear receptors and pattern recognition receptors remains incompletely untangled. Moreover, there is a lack of systematic experimental evidence on how multi−component traditional Chinese medicine formulas can simultaneously modulate the microbiome and bile acid metabolism to alleviate SI−ALI.

Furthermore, in the context of ALI and sepsis triggered by bacterial infection and LPS stimulation, TLR4 recognizes LPS and activates downstream pro-inflammatory cascades via the adaptor MYD88, leading to IκB degradation and NF-κB translocation. This process upregulates inflammatory cytokines such as IL-1β, IL-6, and TNF-α, resulting in alveolar epithelial and vascular endothelial injury, increased permeability, and inflammatory exudation (Zhang et al., 2021; Guo et al., 2024). Concurrently, TLR4 activation also induces phosphorylation of JNK, a member of the MAPK family, which plays a critical role in LPS-induced inflammatory responses and neutrophil function. The TLR4-JNK axis has been demonstrated to determine the occurrence and extent of neutrophil NETosis upon LPS stimulation (Khan et al., 2017). The reviews and experimental evidence have extended and confirmed this mechanism. LPS activates TLR4, which subsequently triggers NADPH oxidase 2 (NOX2)-dependent oxidative stress via c-Jun N-terminal kinase (JNK) signaling(Wang H, et al., 2024; Espiritu and O’Sullivan, 2025), thereby promoting the release of neutrophil extracellular traps (NETs). In sepsis models, blocking TLR4 or inhibiting JNK significantly reduces NET formation and tissue injury (Xia et al., 2024).Studies have shown that MYD88 drives detrimental intravascular NET formation in sepsis and exacerbates disease progression; MYD88 deficiency significantly reduces lethal NET formation and improves outcomes, indicating that MYD88 is a key upstream regulator of excessive NET activation (Englert et al., 2025). Therefore, in the pathogenesis of SI-ALI, TLR4/MYD88-mediated NF-κB and JNK signaling not only amplify the pro-inflammatory cytokine storm but also promote NET generation and oxidative stress, collectively contributing to endothelial dysfunction and lung injury. Blocking this pathway effectively alleviates inflammation and lung injury, underscoring its significance as a therapeutic target.

The reviews and methodological evaluations have indicated that although existing clinical studies on TCM for sepsis have limitations in randomization and sample size, a growing body of animal model research and increasingly standardized clinical trials are demonstrating the potential effects of TCM compound formulations in improving sepsis−related organ function, alleviating inflammation, and restoring the intestinal barrier. This suggests that this field is entering a phase of more rigorous clinical validation (Fu et al., 2024; Ji et al., 2025).Preclinical studies suggest that the therapeutic effect of MDD on sepsis may be achieved through synergistic regulation of pulmonary and gastrointestinal functions (Chen et al., 2016, 2018). This finding provides modern clinical evidence for the classical theory of TCM that “the lung and intestine are interiorly-exteriorly related.” It indicates that this formula can exert favorable comprehensive therapeutic effects via the approach of “simultaneous treatment of the lung and intestine,” as demonstrated in years of clinical practice at Guangdong Provincial Hospital of Chinese Medicine and Guizhou Provincial Hospital of Traditional Chinese Medicine, with its protective effect on gastrointestinal function being particularly notable (Chen et al., 2016, 2018). The composition of MDD includes *Rheum palmatum L* (Dahuang), Na_2_SO_4_·10H_2_O (Mangxiao), *Magnolia officinalis* var. *biloba* (Houpo), *Forsythia suspensa* (Lianqiao), *Scutellaria baicalensis Georgi* (Huangqin), *Prunus armeniaca* var. *armeniaca* (Xingren), *Bletilla striata* (Baiji), and *Panax notoginseng* (Sanqi). The plant names have been checked with http://www.worldfloraonline.org (04/09/2025).

The major constituents of MDD have been reported to be involved in sepsis, acute lung injury, and the regulation of intestinal microbiota. Further microbiome and metabolome studies have demonstrated that baicalin alleviates intestinal inflammation by modulating the composition of the gut microbiota and enhancing intestinal barrier function, which complements its effects on inhibiting inflammatory pathways and promoting the polarization of macrophages toward an anti-inflammatory phenotype (Wan et al., 2024; Zhang et al., 2025). Emodin has been reported to alleviate LPS-induced lung injury by inhibiting the JNK/Nur77/c-Jun pathway and to ameliorate sepsis-associated lung injury both *in vitro* and *in vivo* through modulation of SIRT1 (Liu et al., 2022; Xie et al., 2022). Furthermore, honokiol, a major active component of *magnolia officinalis*, significantly alleviates pulmonary edema and histopathological damage, and improves survival rates in LPS- and CLP-induced acute lung injury or sepsis models by inhibiting inflammation and oxidative stress, protecting the pulmonary microvascular endothelial barrier, and suppressing NLRP3-mediated pyroptosis (Weng et al., 2011; Liu et al., 2021, 2023).Ginsenoside-related metabolites are associated with the regulation of immune homeostasis (Zhuang et al., 2021).It is interesting that *Rheum palmatum L*(Dahuang), Na_2_SO_4_·10H_2_O(Mangxiao), *Magnolia officinalis* var. *biloba*(Houpo) improve ALI by regulating the expression of HIF-1α, downregulating glycolysis, and reducing inflammation (Shang et al., 2024). Besides, multiple major components in MDD have been reported to modulate the gut microbiota. Baicalin can alleviate intestinal inflammation and improve intestinal barrier function by inhibiting inflammatory pathways and remodeling the gut microbiota composition (Zhang S, et al., 2024).Emodin significantly alters the structure of the gut microbial community, reduces the Firmicutes/Bacteroidetes ratio, and promotes the production of beneficial metabolites, thereby ameliorating inflammation and intestinal permeability (Mabwi et al., 2023).Ginsenosides simultaneously increased short-chain fatty acids (SCFAs) and significantly modulated bile acids in the blood and tissues, with changes in the microbiota being correlated with alterations in bile acids (Li et al., 2025). The study demonstrated that ginsenoside Rk3 enriched butyrate-producing bacteria and elevated butyrate levels. As a key microbial metabolite, butyrate mediated the anti-inflammatory and neuroprotective effects (Zhou S, et al., 2025).Honokiol has been shown to ameliorate non-alcoholic fatty liver disease in mice, accompanied by remodeling of the ileal gut microbiota and alterations in the serum bile acid profile; notably, significant correlations between microbial taxa and bile acid levels suggest that honokiol exerts its protective effects through coordinated regulation of gut microbiota and bile acid metabolism (Zhai et al., 2023). Therefore, the multi-component combination of MDD possesses the potential to simultaneously target the gut microbiota ecosystem, bile acid metabolism, and pulmonary cell signaling pathways, which constitutes the theoretical basis for selecting MDD as an interventional agent.

This study systematically analyzed the gut microbiota characteristics and associated bile acid profiles in the control group, model group, and MDD group, aiming to investigate how gut microbiota and its mediated bile acid metabolic disorders influence systemic inflammation and SI-ALI. The results are intended to elucidate the microbiological and metabolomic basis of the TCM principle of “simultaneous treatment of lung and gut” in SI-ALI. We hypothesized that MDD alleviates SI-ALI by modulating the gut microbiota–bile acid metabolism, activating the FXR, thereby inhibiting the TLR4/MYD88 pathway, reducing NETs formation, and protecting the pulmonary vascular endothelium. These findings provide a theoretical foundation for developing novel therapeutic strategies targeting the microbiota-bile acid axis.

## 2 Materials and methods

### 2.1 MDD formulation preparation

The constituents of MDD were obtained from the Traditional Chinese Medicine pharmacy of Guangdong Provincial Hospital of Traditional Chinese Medicine, with Dahuang dosed at 20g and all other herbs at 10g each. Detailed information can be found in **Table 1**. To prepare the decoction, 500 mL of water was added to the herbs and boiled until the solution was concentrated to 50 mL, yielding a final crude herb concentration of 1.8 g/mL. In the clinical protocol, a patient weighing 60 kg received a dose corresponding to 90g of raw herbs. The herbs were decocted with 500 mL of water and concentrated to yield 4.5 g of extract. Therefore, the clinical dose was equivalent to 4.5g/60 kg. First, based on the body surface area conversion principle, this human dose was converted to the mouse equivalent dose, resulting in 0.92 g/kg. This dose was subsequently designated as the medium dose. On this basis, 0.5 times (0.46 g/kg) and 2.0 times (1.84 g/kg) of this dose were defined as the low and high doses, respectively. For the positive control group, dexamethasone (DEX) tablets (Xianju, LB2426) were used to prepare a solution with a concentration of 0.5 mg/mL.

**Table 1:** Component herbs of MDD.

Latin name	Chinese name	Place of origin	Manufacturers	Batch number
Rheum palmatum L	Da Huang	Gansu Province, China	Sinopsin Group Feng Liaoxing (Foshan) Pharmaceutical Co., LTD	C12405083
–	Mangxiao	Sichuan Province,China	Kangmei Pharmaceutical Co., LTD	240200961
Houpoea officinalis	Houpo	Guangxi Province,China	Yulin Bencaotang Chinese Medicine Co., LTD	240501
Forsythia suspensa	Lianqiao	Shanxi Province,China	Sinopsin Group Feng Liaoxing (Foshan) Pharmaceutical Co., LTD	C22403120
Scutellaria baicalensis Georgi	Huangqin	Shanxi Province,China	Lingnan Traditional Chinese Medicine Tablets Co. LTD	2405001
Semen Armeniacae Amarum	Xingren	Hebei Province, China	Kangmei Pharmaceutical Co., LTD	240502301
Bletilla striata	Baiji	Yunnan Province, China	Sinopsin Group Feng Liaoxing (Foshan) Pharmaceutical Co., LTD	C12312080
Panax notoginseng	Sanqi	Yunnan Province, China	Lingnan Traditional Chinese Medicine Tablets Co. LTD	2403003

**MDD,** Modified Dachengqi Decoction.

### 2.2 Ultra performance liquid chromatography mass spectrometry analysis of MDD

A total of 6 mL of the prepared MDD solution was aliquoted into three samples of 2 mL each for triplicate testing. The analysis was conducted using a Vanquish ultra-high-performance liquid chromatography (UHPLC) system from Thermo Fisher Scientific, employing a Phenomenex Kinetex C18 column (2.1 mm × 100 mm, 2.6 μm) for chromatographic separation of the target compounds. The mobile phase A consisted of water with 0.01% acetic acid, while phase B was a mixture of isopropanol and acetonitrile (1:1, v/v). The sample tray temperature was maintained at 4°C, and the injection volume was set at 2 μL. The Orbitrap Exploris 120 mass spectrometer was operated under the control of the Xcalibur software (version 4.4, Thermo Fisher Scientific) for the acquisition of both MS and MS/MS data. The detailed parameters were as follows: Sheath gas flow rate: 50 Arb, Aux gas flow rate: 15 Arb, Capillary temperature: 320 °C, Full ms resolution: 60000, MS/MS resolution: 15000, Collision energy: SNCE 20/30/40, Spray Voltage: 3.8 kV (positive) or -3.4 kV (negative).

### 2.3 Animal experiments

#### 2.3.1 Dosage of treatment

Eight-week-old male *C57BL/6* mice (n=190) were housed at the Guangdong Provincial Academy of Chinese Medical Sciences, and all experimental procedures were conducted in accordance with the ARRIVE 2.0 guidelines (Percie et al., 2020)under certificate number No. 2024017 (obtained March 18, 2024). After a one-week acclimatization period, the C57BL/6 mice were randomly grouped using a random number table into the control group (CON), lipopolysaccharide group (LPS), low-dose of MDD group (MDD-L), medium-dose of MDD group (MDD-M), high-dose of MDD group (MDD-H), and dexamethasone group (DEX). Two days before establishing the model, we administered preventive treatment. In addition to CON and LPS groups, the remaining groups received the corresponding intervention. The administered doses of MDD were 0.46 g/kg (low dose), 0.92 g/kg (intermediate dose), and 1.84 g/kg (high dose), while the dose of dexamethasone was 5 mg/kg (Son et al., 2022).

#### 2.3.2 Sepsis mortality

**Figure 1:**
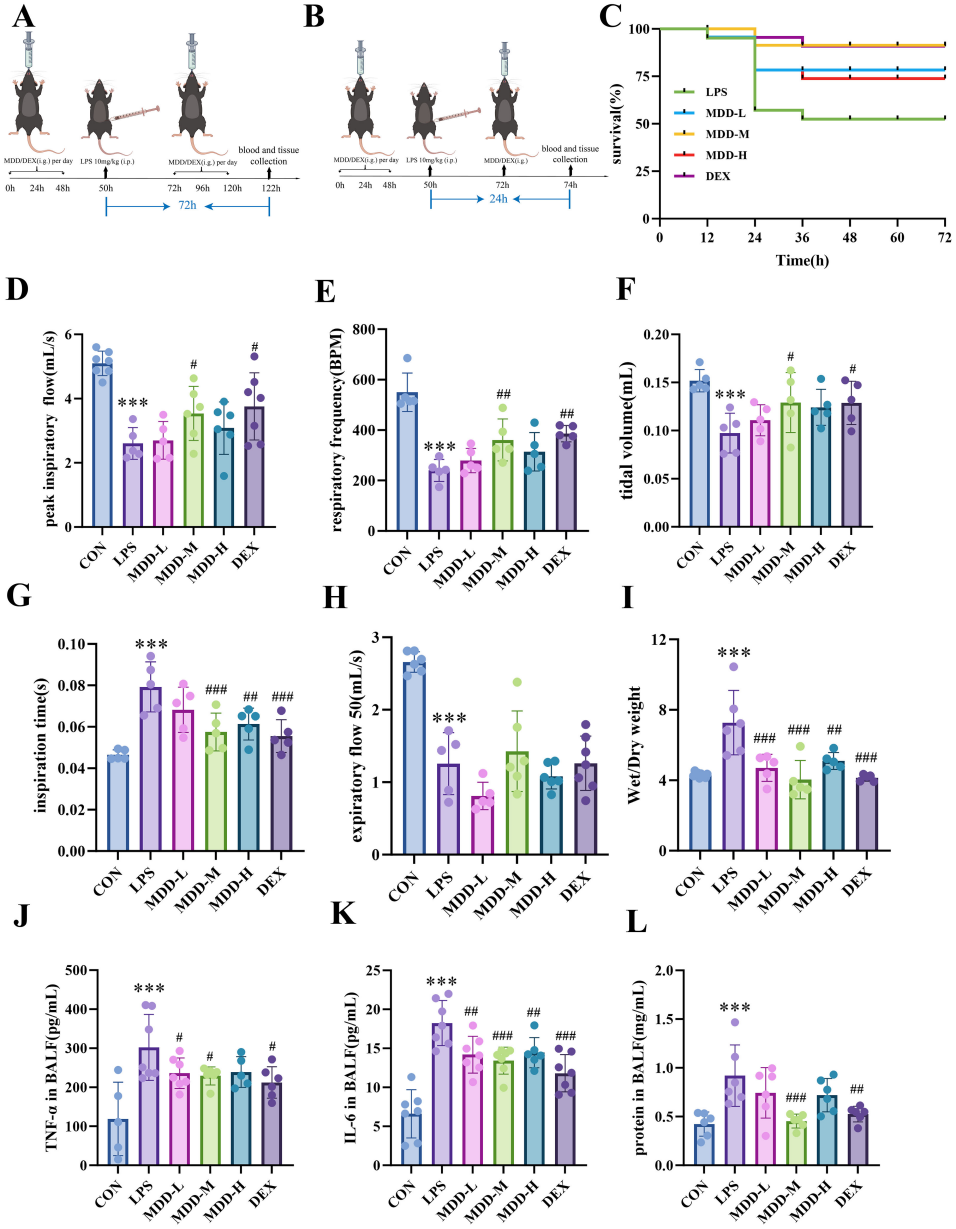
MDD can reduce mortality in mice with SI-ALI and alleviate inflammation and respiratory function impairment. **(A)** Experimental protocol for SI-ALI model and treatment to 72h survival and **(B)** 24h-treatment; **(C)** Survival Rate of SI-ALI(n=10); **(D)** peak inspiratory flow(mL/s); **(E)** respiratory frequency(BPM)(n=5-7); **(F)** tidal volume(mL)(n=5); **(G)** inspiration time(s)(n=5); **(H)** expiratory flow 50(mL/s)(n=5-7); **(I)** Wet/Dry (W/D) ratio of the lungs(n=5-6); **(J-L)**The concentrations of TNF-α, IL-6, and protein in BALF(n=5-7). These results were presented as mean ± SD. *p < 0.05, **p < 0.01,***p < 0.001, compared with the CON group. #*p* < 0.05, ##*p* < 0.01, ###*p* < 0.001, compared with the LPS group.

In this study, 100 mice were initially used to observe the 72-hour mortality rate of sepsis. The experimental animals were randomly assigned to the following five groups (n=20 per group): the LPS group, MDD-L group, MDD-M group, MDD-H group, and the DEX group. Two days after continuous MDD and DEX gavage, the mice were intraperitoneally injected with LPS (Sigma, L2880-100MG) to establish a sepsis model. LPS was diluted with physiological saline to a concentration of 1 mg/mL and injected into the abdominal cavity at a dosage of 10 mg/kg (Park et al., 2019).Subsequently, the mouse mortality was recorded every 12 hours for 72 hours. Surviving mice received a daily oral gavage during this period. The final mortality rate for each group is shown in **Figure 1A**.

#### 2.3.3 Study on the mechanism of MDD

To assess disease severity, we selected the critical time point of 24 hours post-LPS injection for observation, with a sample size of 15 mice per group across six groups: the CON group, LPS, MDD-L, MDD-M, MDD-H, and DEX groups. Consistent with the above experiment, we pretreated mice in the corresponding groups with MDD and DEX for 2 consecutive days before LPS injection. At 24 hours post-LPS injection, we examined the lung function of the mice and collected tissues for subsequent analysis (**Figure 1B**).

### 2.4 Assessment of respiratory function

Pulmonary function assessments were conducted 24 hours after LPS injection to evaluate the severity of lung injury. Mice were placed in the EMMSlink Whole Body Plethysmography system (EMMSlinks WBP) and allowed to acclimate before measurements commenced. Each mouse underwent a 5-minute assessment period.

### 2.5 Tissue collection

Following the respiratory function assessments, three fecal pellets were collected from each mouse. Subsequently, the mice were anesthetized for blood collection, and bronchoalveolar lavage was performed on the right lung with saline to obtain bronchoalveolar lavage fluid (BALF). The remaining lung tissue alongside intestinal tissue was harvested and stored at -80°C for future analysis.

### 2.6 Lung wet-to-dry weight ratio

The wet-to-dry weight ratio of lung tissues is commonly utilized to assess the severity of pulmonary edema. In brief, the right lung lobes were weighed immediately after surface fluids were removed to obtain the wet weight. Subsequently, the lung tissues were baked at 60°C until a constant weight was achieved to determine the dry weight. Ultimately, the wet-to-dry lung weight ratio was calculated to evaluate the severity of edema.

### 2.7 Histopathological staining

Lung and intestinal tissues were sectioned into 4 µm-thick slices after fixation in tissue fixative, dehydration and embedding. The sections were then stained with hematoxylin and eosin (H&E), and examined under a microscope. H&E stained slides were scored as previously describe (Matute-Bello et al., 2011; Chassaing et al., 2015).

### 2.8 BALF preparation and analysis

The previously collected BALF was centrifuged at 1500 rpm for 10 minutes, and the supernatant was obtained for protein content analysis using the BCA kit (P0011, Beyotime). The levels of TNF-α and IL-6 in BALF were detected by using the Mouse TNF-α ELISA kit (ZC-39024, Zhuocai) and Mouse IL-6 ELISA Kit (ZC-37988, Zhuocai) in accordance with the manufacturer’s instructions. Finally, the concentrations of TNF-α and IL-6 were calculated through standard curves.

### 2.9 16S rRNA microbial community analysis

Fecal samples were collected from mice in the CON, LPS, and MDD-M groups, with each group comprising six specimens, for 16S rRNA analysis.

#### 2.9.1 DNA extraction and amplification

Total genomic DNA was extracted using MagPure Soil DNA LQ Kit (Magan) following the manufacturer’s instructions. DNA concentration and integrity were measured with NanoDrop 2000 (Thermo Fisher Scientific, USA) and agarose gel electrophoresis. Extracted DNA was stored at -20 °C until further processing. The extracted DNA was used as a template for PCR amplification of bacterial 16S rRNA genes with the barcoded primers and Takara Ex Taq (Takara). For bacterial diversity analysis, V3-V4 variable regions of 16S rRNA genes were amplified with universal primers 343F (5’-TACGGRAGGCAGCAG-3’) and 798R (5’-AGGGTATCTAATCCT-3’) and 907R (5’-CCGTCAATTCMTTTRAGTTT-3’) (Xiong et al., 2012).

#### 2.9.2 Library construction and sequencing

The Amplicon quality was visualized using agarose gel electrophoresis. The PCR products were purified with AMPure XP beads (Agencourt) and amplified for another round of PCR. After being purified with the AMPure XP beads again, the final amplicon was quantified using Qubit dsDNA Assay Kit (Thermo Fisher Scientific, USA). The concentrations were then adjusted for sequencing. Sequencing was performed on an Illumina NovaSeq 6000 with 250 bp paired-end reads (Illumina Inc., San Diego, CA; OE Biotech Company; Shanghai, China).

#### 2.9.3 16S rRNA amplicon sequencing analysis process

The library sequencing and data processing were conducted by OE Biotech Co., Ltd. (Shanghai, China). Raw sequencing data were in FASTQ format. Paired-end reads were then preprocessed using Cutadapt software to detect and cut off the adapter. After trimming, paired-end reads were filtering low quality sequences, denoised, merged and detect and cut off the chimera reads using DADA2 (Callahan et al., 2016)with the default parameters of QIIME2 (Bolyen et al., 2019) (2020.11). At last, the software output the representative reads and the ASV abundance table. The representative read of each ASV was selected using the QIIME2 package. All representative reads were annotated and blasted against the SILVA database (Version 138) using q2-feature-classifier with the default parameters.

QIIME2 software was used for alpha and beta diversity analysis. The microbial diversity in samples was estimated using alpha diversity metrics, including the Chao1 index (Chao and Bunge, 2002) and Shannon index (Hill et al., 2003). The unweighted Unifrac distance matrix performed by R package was used for unweighted Unifrac Principal coordinates analysis (PCoA) to estimate the beta diversity. Next, the R package was used to analyze the significant differences between different groups using the Kruskal-Wallis statistical test. The linear discriminant analysis effect size (LEfSe) method was used to compare the taxonomy abundance spectrum.

### 2.10 Targeted bile acid metabolism

Fecal samples were collected from the CON, LPS, and MDD-M groups for the assessment of targeted bile acid metabolism, with each group comprising six samples. An analytical balance was used to accurately measure the required amount of the standard compound, which was then dissolved in water to prepare the standard stock solution at a concentration of 1 mg/mL. The standard working solution was subsequently prepared from the standard stock solution, and a standard curve was generated through serial dilution of the mixed standard solution. Standard information and methodological Data are provided in **Supplementary File 1-2**.

#### 2.10.1 Sample pretreatment

An appropriate amount of fecal samples was taken, accurately weighed, and recorded. The samples were then loaded into pre-cooled 1.5 mL Eppendorf (EP) tubes (pre-cooled at -20 °C). Subsequently, 10 μL of a mixed internal standard solution (comprising CA-d4, Lyso PC17, GCA-C13, and L-2-chlorophenylalanine) was added, followed by the addition of 590 μL of methanol containing 1 mM butylated hydroxytoluene (BHT). Two steel balls were added to each sample, and the samples were swirled for 30 seconds. The samples were then placed in a -20 °C refrigerator for 2 minutes, after which they were ground in a grinder operating at 60 Hz for 1 minute. The samples were subjected to ultrasonic extraction in an ice bath for 10 minutes and subsequently centrifuged for 10 minutes at 4 °C and 12,000 rpm. Following centrifugation, 200 μL of the supernatant was carefully aspirated using a syringe and filtered through a 0.22 μm organic phase pinhole filter. Finally, the filtered samples were transferred to brown sample bottles and stored at -80 °C until further machine analysis.

#### 2.10.2 Chromatography and mass spectrometry

In this study, the UPLC-ESI-MS/MS analytical method was employed for both the qualitative and quantitative detection of target metabolites. The specific analytical conditions and methodologies utilized are detailed as follows:

#### 2.10.3 Chromatographic methods

The sample volume utilized was 5 μL, with a flow rate of 0.45 mL/min. The mobile phases employed were as follows: Phase A consisted of a 0.1% formic acid aqueous solution, while Phase B comprised a mixture of methanol, acetonitrile, and isopropanol in a 1:1:1 ratio, also containing 0.1% formic acid. The gradient elution procedure was executed according to the following schedule: 0 min, A/B (80:20, v/v); 0.5 min, A/B (80:20, v/v); 1.5 min, A/B (62:38, v/v); 12 min, A/B (50:50, v/v); 17.5 min, A/B (5:95, v/v); 19 min, A/B (5:95, v/v); 19.01 min, A/B (80:20, v/v); and 20 min, A/B (80:20, v/v).

#### 2.10.4 Mass spectrometry

The mass spectrometry analysis was conducted under the following conditions: the gas curtain was maintained at a pressure of 35 psi, and the collision-activated dissociation (CAD) parameters were set to medium. The negative ion spray voltage was -4500 V, while the positive ion spray voltage was 5500 V. The ion source temperature was regulated at 450°C, and the column temperature was maintained at 45 °C. Besides, the spray gas (Gas1) and the auxiliary heating gas (Gas2) were both set to a pressure of 55 psi.

#### 2.10.5 Qualitative and quantitative analysis

Targeted metabolites were analyzed in multiple reaction monitoring (MRM) mode. The MRM pairs, declustering potentials (DP), and collision energies (CE) were optimized for each analyte. Data acquisitions and further analysis were conducted using Analyst software. SCIEX OS-MQ software was used to quantify all metabolites.

### 2.11 Correlation analysis between gut microbiota and bile acid metabolite

The data on microbial diversity and the metabolome were initially screened based on the original differential criteria. Subsequently, the correlation coefficients among all differential features were calculated using the Spearman correlation algorithm.

### 2.12 Immunofluorescence staining

After fixation, the lung tissues were embedded and then sectioned. The sections were deparaffinized, dehydrated, and restored with a citrate-EDTA antigen retrieval solution. The primary antibodies, VCAM-1 (1:200, ab134047, Abcam), VE-cadherin (1:200, 27956-1-AP, Proteintech), CitH3 (1:200, 97272S, CST), MPO (1:200, AF3667, R&D),phospho-SAPK/JNK(1:200,9255,CST), and phospho-p65(1:500,3033,CST)were utilized for overnight incubation with the lung sections at 4°C. Afterward, the sections were exposed to fluorescent-conjugated secondary antibodies for 2 h at room temperature in darkness. After three washes with PBS, the sections were stained with DAPI solution (Beyotime, Shanghai, China). Ultimately, images were captured employing a fluorescence microscope.

### 2.13 ELISA assay

The concentration of MPO-DNA complexes in mouse serum was measured using the Mouse MPO-DNA ELISA Kit (ZC-56424, Zhuocai).

### 2.14 Immunohistochemical staining

Lung and intestinal tissue samples were fixed in 4% paraformaldehyde, paraffin-embedded, and sectioned (4 μm). After deparaffinization and rehydration, antigen retrieval was performed in a citrate buffer at 95 °C for 20 minutes. Endogenous peroxidase was blocked with 3% H_2_O_2_ for 10 minutes, and nonspecific binding was blocked with 5% BSA for 30 minutes. Sections were incubated with primary FXR (1:200, ab155124, Abcam) antibody at 4 °C overnight, followed by HRP-conjugated secondary antibody at room temperature for 1 hour. DAB was used for chromogenic development, and hematoxylin was applied for counterstaining. After dehydration and mounting, FXR expression and localization were observed under a microscope. The positive area was quantitatively analyzed and calculated using ImageJ software.

### 2.15 Western blotting analysis

Lung tissue lysates with approximately 90 μg of proteins were resolved on 10% SDS-PAGE and were subjected to western blot assay utilizing the anti-CitH3 (1:1000, 97272S, CST), PAD4 (1:1000, HA721657, HUABIO), TLR4 (1:1000, 66350-1-Ig, Proteintech), GAPDH(1:3000,60004-1-Ig,Proteintech), β-actin(1:3000,66009-1-Ig,Proteintech), β-tubulin(1:500,ab18207,Abcam), MYD88 (1:1000, ab219413, Abcam). After incubation with the appropriate secondary antibody, the bands were visualized with the Molecular Imager System (BIO-RAD, Hercules, USA) with an enhanced chemiluminescence substrate (Thermo Fisher Scientific, Massachusetts, USA). Subsequently, the intensities of these bands were quantitatively analyzed and calculated using ImageJ software for precise and accurate measurements.

### 2.16 Network pharmacological analysis

#### 2.16.1 Screening of core drug active ingredients and targets

Based on data mining, rhubarb, mirabilitum, magnolia bark, forsythia, scutellaria, almond, bletilla striata, and pseudo-ginseng were selected as the core drugs. Their chemical components were retrieved from the TCMSP database, and active ingredients with an oral bioavailability (OB) greater than 30% and a drug-likeness index (DL) of 0.18 or higher were screened. Effective protein targets were obtained using TCMSP, and the target names were standardized through the UniProt database.

#### 2.16.2 Acquisition of sepsis targets

Using “sepsis” as the keyword, genes related to sepsis were searched in the TTD, OMIM, DrugBank, and GeneCards databases. The gene names were then normalized and deduplicated through the UniProt database.

#### 2.16.3 Construction of PPI network and “drug-ingredient-target” network

The intersection of genes related to core drug targets and sepsis targets was obtained using Jvenn. These genes were imported into the String database for protein-protein interaction (PPI) analysis (confidence level > 0.9, species “*Homo sapiens*”), and the results were visualized using Cytoscape 3.9.1. A “core drug-active ingredient-intersecting target” network was constructed, and the network topology parameters were analyzed using the CytoNCA plugin to screen for key hub targets (DC, BC, CC greater than the median).

#### 2.16.4 Functional enrichment analysis

Metascape (http://metascape.org) was employed to perform Gene Ontology (GO) annotation and Kyoto Encyclopedia of Genes and Genomes (KEGG) pathway enrichment analysis on the intersecting targets (*P* < 0.01). The GO analysis included three categories: Biological Process (BP), Cellular Component (CC), and Molecular Function (MF). Enriched terms with *P* < 0.01 and containing at least three genes were selected for further analysis. The significance threshold for enrichment was set as a *P*-value < 0.01 and a minimum enrichment factor of 1.5. Redundant terms were removed using Metascape’s built-in redundancy reduction algorithm with a similarity cutoff of 0.3. The top 10 significantly enriched GO terms and KEGG pathways were visualized using an online microbiology tool (https://www.bioinformatics.com.cn). The criteria for selecting representative pathways were based on both statistical significance (*P* < 0.01) and biological relevance to sepsis and lung injury mechanisms.

### 2.17 Statistical analysis

All data were expressed as mean ± standard deviation. Multivariate differences were evaluated by one-way analysis (ANOVA), followed by Fisher’s LSD test. A *p-*value<0.05 was considered statistically significant. All statistical analyses were performed using GraphPad Prism 10.0 (GraphPad Software Inc.).

## 3 Results

### 3.1 Chemical composition of MDD

**Figure 2:**
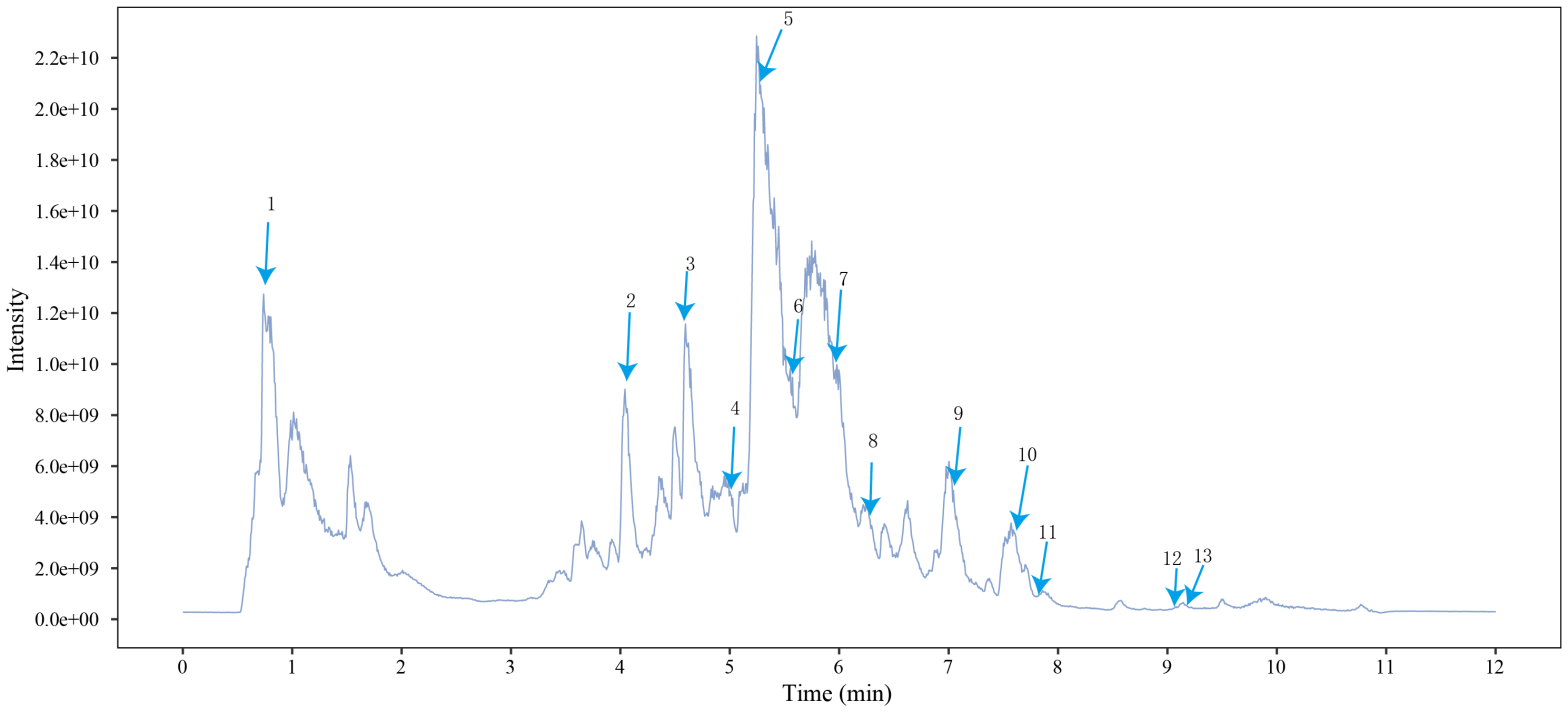
Total ion chromatogram of Modified Dachengqi Decoction (MDD) in negative ion mode of UPLC-MS. 1, 4-Aminobutyric acid (GABA); 2, Amygdalin; 3, Forsythiaside; 4, Scutellarin; 5, Baicalin; 6, Quercetin; 7, Emodin-8-glucoside; 8, batatasin III; 9, Rhein; 10, Emodin; 11, Ginsenoside Rg3; 12, Myristic acid; 13, Linoleic acid.

The chemical composition of MDD was analyzed applying UPLC-MS to establish quality control by identifying the ingredients. A total of 1259 components were identified (**Supplementary File 3**), including Baicalin, 4-aminobutyric acid (GABA), quercetin, emodin, and ginsenoside Rg3, among others. They mainly belong to Anthraquinones, anthrones, Flavones and fatty acids. The results of the negative ion flow chromatography for the MDD formula are presented in **Figure 2**, while **Table 2** lists the 13 compounds along with their basic formulas and structures. Among the identified compounds, 12 components achieved a qualitative level of Level 1, while 1 is Level 2, signifying a high degree of confidence in the identification of these compounds.

**Table 2:** Results of UPLC-MS analysis of MDD.

Peak name	Retention time(min)	m/zmed	ms2Adduct	Formula	Subclass	ms2 name	Level	Chemical structure
1	0.716666667	102.056	[M-H]-	C4H9NO2	Amino acids	4-Aminobutyric acid (GABA)	1	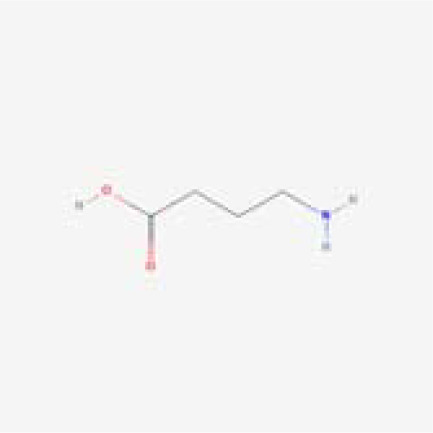
2	4.041666667	456.1506	[M-H]-	C20H27NO11	Cyanogenic glycosides	Amygdalin	1	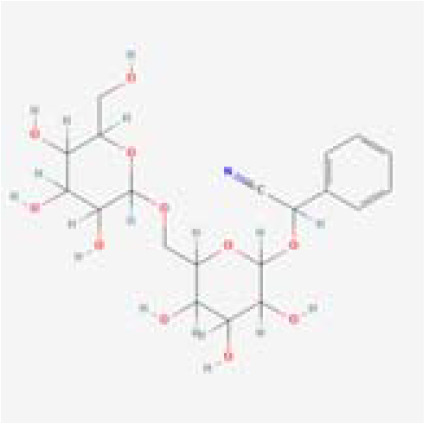
3	4.603333333	623.1975	[M-H]-	C29H36O15	Cinnamic acids and derivatives	Forsythiaside	1	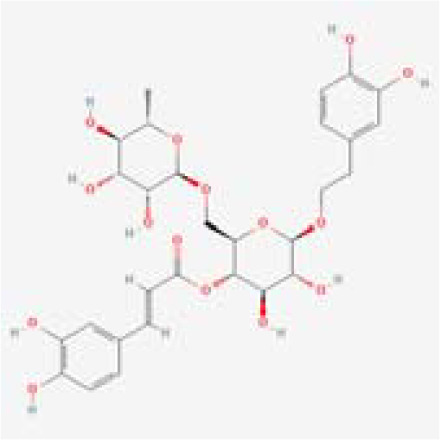
4	4.963333333	461.0725	[M-H]-	C21H18O12	Flavones	Scutellarin	1	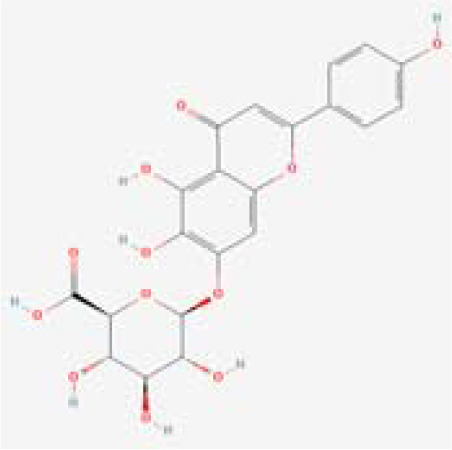
5	5.266666667	445.0768	[M-H]-	C21H18O11	Flavones	Baicalin	1	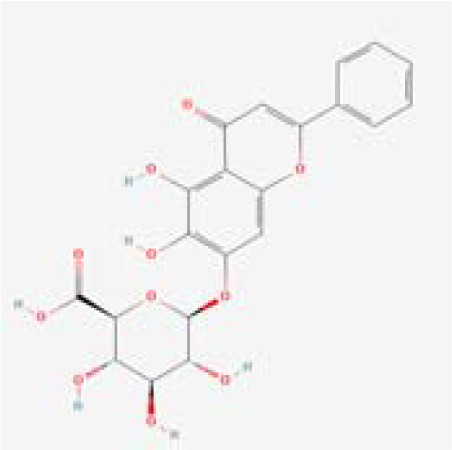
6	5.526666667	301.0352	[M-H]-	C15H10O7	Flavonols	Quercetin	1	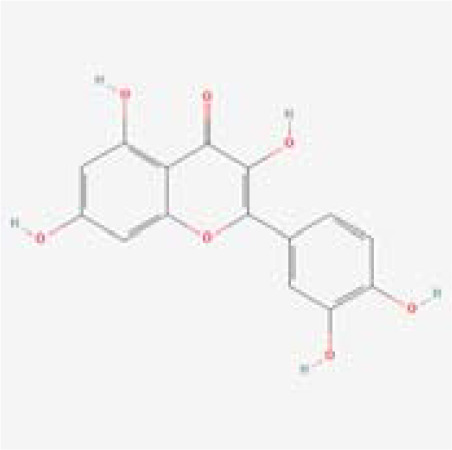
7	5.915	431.0981	[M-H]-	C21H20O10	Anthraquinones and anthrones	Emodin-8-glucoside	1	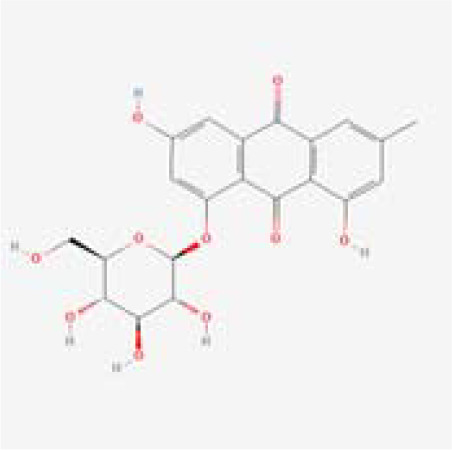
8	6.285	243.1026	[M-H]-	C15H16O3	Monomeric stilbenes	batatasin III	2	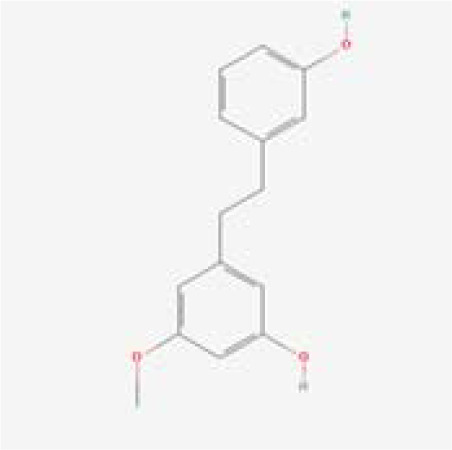
9	7.005	283.0245	[M-H]-	C15H8O6	Anthraquinones and anthrones	Rhein	1	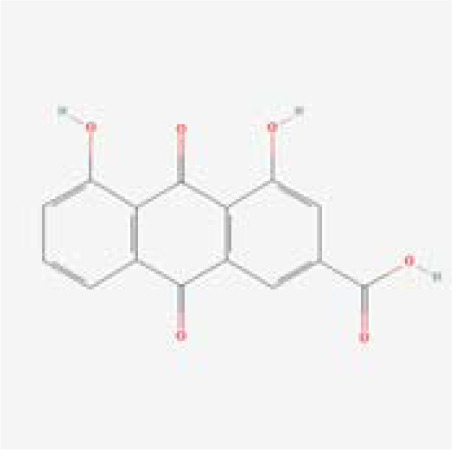
10	7.61	269.0453	[M-H]-	C15H10O5	Anthraquinones and anthrones	Emodin	1	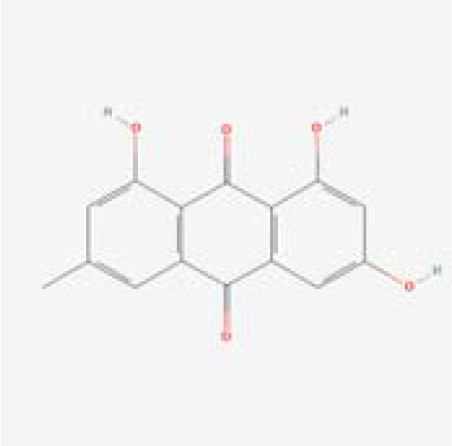
11	7.833333333	783.4899	[M-H]-	C42H72O13	Dammarane and Protostane triterpenoids	Ginsenoside Rg3	1	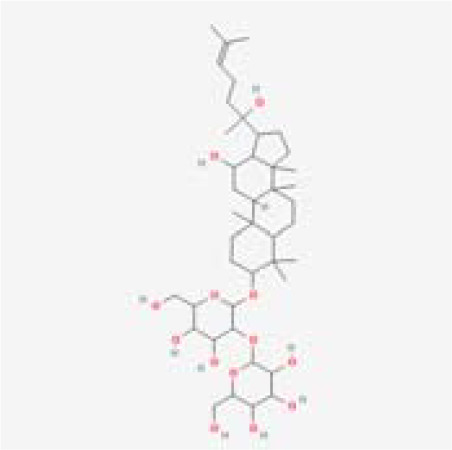
12	9.083333333	227.2017	[M-H]-	C14H28O2	Branched fatty acids	Myristic acid	1	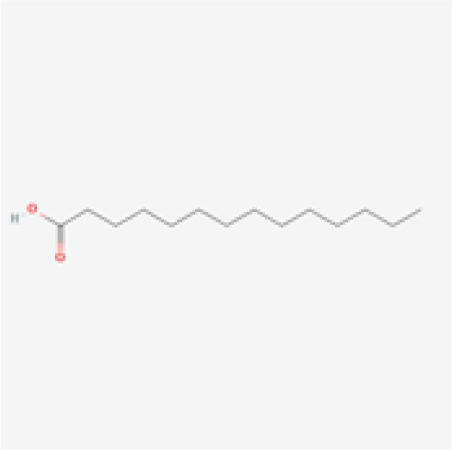
13	9.22	279.233	[M-H]-	C18H32O2	Unsaturated fatty acids	Linoleic acid	1	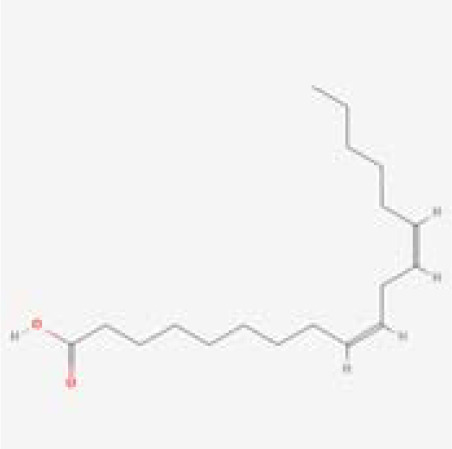

Level 1: Indicates that the sample material’s MS/MS spectrum (all fragment ions of the material) and Retention time match the database material with a score of 0.7 or above.

Level 2: Represents a match between the sample material’s MS/MS spectrum (including all fragment ions) and Retention time with the database material, scoring between 0.5 and 0.7.

### 3.2 MDD lowered sepsis mortality

Sepsis is characterized by a high mortality rate, and survival rates serve as a direct indicator of therapeutic efficacy. Following LPS injection, the survival rate at 72h was 50% (**Figure 1C**). After MDD intervention, the mortality rate markedly decreased, with survival rates of 75%, 90%, and 70% observed in the low, intermediate, and high-dose MDD groups, respectively. Notably, the survival rate in the MDD-M group was comparable to that of the DEX group (90%).

### 3.3 MDD alleviated SI-ALI and improved pulmonary function

Pulmonary function tests reflect the health condition of the lungs, and lung injury typically induces a decline in these indicators. We utilized the EMMSlinks WBP system to assess general parameters, volume indicators, conductance metrics, airway obstruction indicators and ventilation parameters. Compared to the CON group, the LPS group demonstrated a reduction in tidal volume, a decrease in respiratory frequency, and a slowdown in peak inspiratory flow. Besides, general parameters such as inspiration time were prolonged (**Figures 1D–G**). These findings indicate that LPS injection successfully induced SI-ALI, which could be reversed following MDD intervention, with the MDD-M exhibiting the most significant improvement.

However, expiratory flow at 50% (EF50), an indicator of airway obstruction, was significantly reduced in the LPS group. Although MDD intervention showed a trend toward improvement, the difference was not statistically significant (*p* > 0.05) (**Figure 1H**). These findings suggested that MDD can mitigate lung injury caused by sepsis by improving lung volume, gas exchange and ventilation; however, it does not effectively relieve airway obstruction.

Pulmonary edema reflects the extent of lung injury. To assess the pulmonary edema, we measured the W/D ratio of the lungs and found that the LPS group exhibited an increased ratio, while MDD reduced the severity of pulmonary edema (**Figure 1I**). After collecting BALF, we measured the levels of inflammatory factors, such as TNF-α and IL-6, using ELISA. The LPS group showed elevated levels of TNF-α and IL-6, whereas MDD intervention decreased these pro-inflammatory cytokines (**Figures 1J,K**). Moreover, BCA quantification revealed an increase in protein content in the BALF of the LPS group, which was reduced by MDD treatment (**Figure 1L**). In summary, MDD effectively alleviates pulmonary edema, reduces inflammation, and promotes the recovery of respiratory function, with the MDD-M group showing the most significant improvement compared to the DEX group.

### 3.4 MDD reduced inflammatory infiltration in the lungs and intestines, and alleviated endothelial dysfunction in the lungs

**Figure 3:**
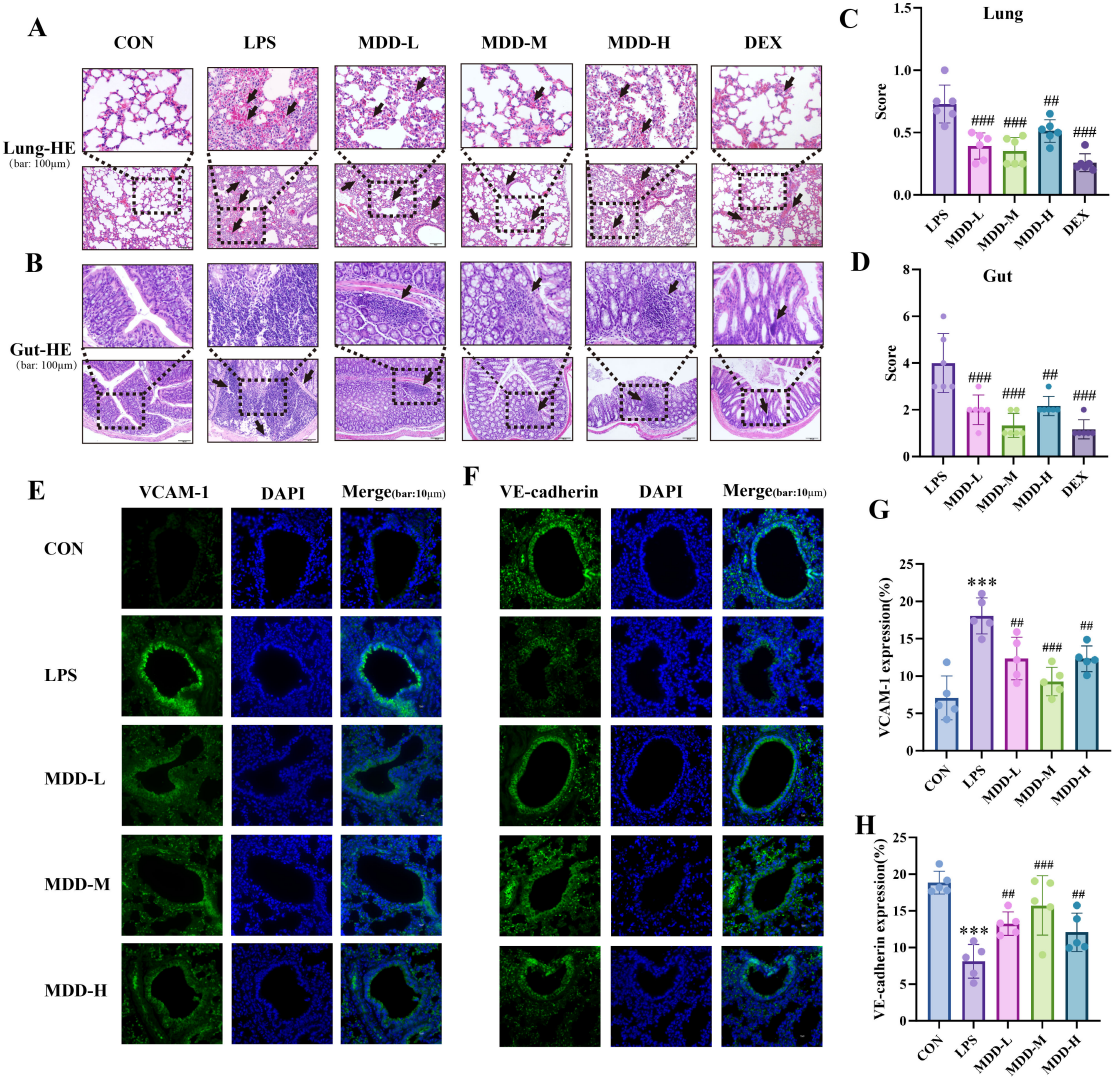
MDD reduced inflammatory infiltration in the lungs and intestine, and alleviated endothelial dysfunction in the lungs. **(A)** H&E staining in the lung and (n=6,bar:100µm)**(B)** gut(n=6,bar:100µm). **(C)** The injury score of lung and **(D)** gut. **(E)** The VCAM-1 and **(F)** VE-cadherin expression in lung tissue(bar:10µm). **(G)** Relative Fluorescence of VCAM-1(n=6) and **(H)** VE-cadherin(n=6) in lung tissue. These results were presented as mean ± SD. *p < 0.05, **p < 0.01,***p < 0.001, compared with the CON group. #*p* < 0.05, ##*p* < 0.01, ###*p* < 0.001, compared with the LPS group.

To further assess pulmonary inflammatory infiltration, we performed H&E staining to observe the extent of inflammatory cell infiltration in the lungs. The LPS group exhibited significant exudation, while the infiltration of inflammatory cells was notably reduced after MDD treatment (**Figure 3A**). Similarly, the LPS group showed marked intestinal injury characterized by extensive inflammatory cell infiltration and destruction of the intestinal wall (**Figure 3B**). However, MDD intervention resulted in marked improvement in both lung and intestinal conditions (**Figures 3C,D**).

One of the hallmarks of sepsis is endothelial dysfunction (Zhang et al., 2023). Given that MDD significantly improved pulmonary exudation, we hypothesized that this effect may be associated with the amelioration of pulmonary vascular endothelial dysfunction. We next assessed the expression of vascular cell adhesion molecule-1(VCAM-1) and vascular endothelial cadherin (VE-cadherin), and found that in the LPS group, the endothelial integrity marker VE-cadherin (Giannotta et al., 2013) was reduced (**Figure 3E**), while the endothelial injury marker VCAM-1 (Wang L. et al., 2024) was elevated (**Figure 3F**). Notably, MDD treatment alleviated the severity of endothelial dysfunction (**Figures 3G, H**). Collectively, these findings suggest that MDD alleviates SI-ALI by enhancing endothelial function.

### 3.5 MDD improved gut dysbiosis

**Figure 4:**
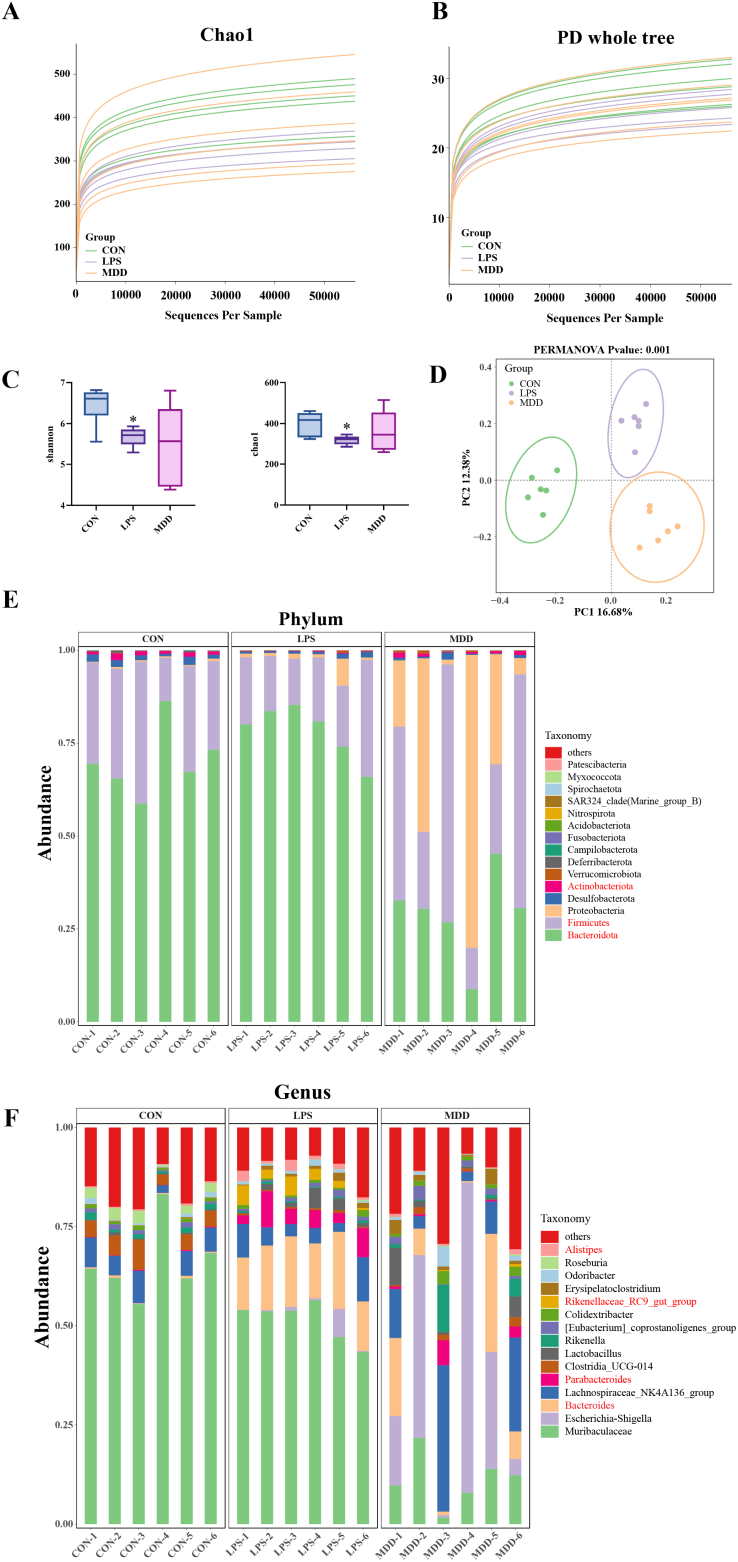
MDD improved the dysbiosis of the gut microbiota. **(A)** The Chao1 and **(B)** PD whole tree curves. **(C)** α-diversity analysis based on the Shannon index(n=6) and Chao1 index(n=6). **(D)** PCoA plots of β-diversity(n=6). **(E)** Bacterial taxa profiling of gut microbiota at the phylum level. **(F)** Bacterial taxa profiling of gut microbiota at the genus level. *p < 0.05, **p < 0.01,***p < 0.001, compared with the CON group. #*p* < 0.05, ##*p* < 0.01, ###*p* < 0.001, compared with the LPS group.

TCM emphasizes the connection between the lungs and intestine, which has attracted significant interest in recent years (Wang et al., 2022, 2023). Our results demonstrated that MDD confers protection against intestinal injury, potentially through the restoration of gut microbiota dysbiosis. Analysis of 16S rRNA sequencing data revealed that as the sampling depth increased, the Chao1 and phylogenetic diversity (PD) whole tree curves plateaued, indicating sufficient sequencing depth for reliable downstream analysis (**Figures 4A, B**).

The α-diversity analysis validated that the Chao1 and Shannon indices in the LPS were significantly lower than those in the CON group, suggesting that LPS markedly reduced the gut microbiota diversity. In contrast, these indices were higher in the MDD group than in the LPS group. However, no significant difference was observed between the two groups (**Figure 4C**).

The PCoA analysis revealed distinct β-diversity clustering patterns among the CON, LPS and MDD groups (**Figure 4D**). Notably, the clustering trend of the MDD was similar to that of the CON group, indicating shared characteristics in their gut microbiota. These results suggest that the improvement of SI-ALI by MDD may be attributed to some extent to the modulation of gut microbiota composition. Next, we investigated the specific taxonomic alterations in gut microbiota induced by MDD.

At the phylum level, *Actinobacteriota*, *Firmicutes* and *Bacteroidota* were found to play significant regulatory roles (**Figure 4E**). Compared to the CON group, the abundance of *Actinobacteriota* and *Firmicutes* in the LPS was comparatively reduced, while MDD reversed this trend. On the contrary, the abundance of *Bacteroidota* was relatively higher in the LPS group, but MDD markedly reversed this increase in abundance.

Next, we investigated the differences in microbial communities at the genus level. The results revealed that compared to the CON group, the abundance of *Alistipes*, *Rikenellaceae_RC9_gut_group*, *Parabacteroides* and *Bacteroides* was increased in the LPS group, while MDD reversed this trend (**Figure 4F**). These findings confirm that MDD plays an important role in multiple levels of the gut microbiota within the SI-ALI model.

### 3.6 MDD regulated bile acid metabolism

Bile acids, as crucial metabolites of gut microbiota, can regulate the severity of SI-ALI based on their concentration (He et al., 2023). Studies have demonstrated that bile acids exhibit concentration-dependent immunomodulatory properties. In acute pancreatitis models, conjugated bile acids such as GCDCA significantly suppress NLRP3 inflammasome activation by activating the TGR5 receptor pathway (Zhang Z. Y. et al., 2024). However, at high concentrations, bile acids exhibit cytotoxicity, Deoxycholic acid (DCA) and CDCA were found to induce robust secretion of pro-inflammatory cytokines IL-1α and IL-1β from bone marrow-derived dendritic cells in ex vivo experiments (Oleszycka et al., 2023).

**Figure 5:**
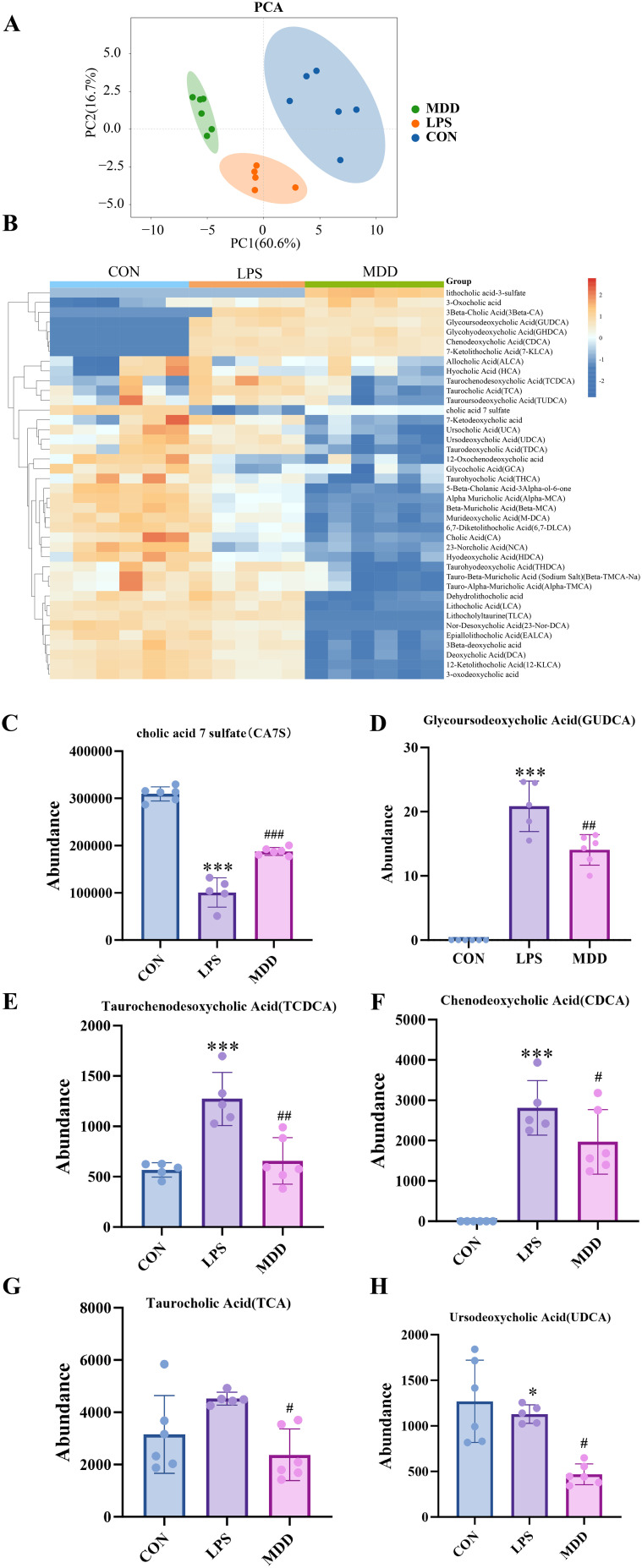
MDD regulates bile acid metabolism. **(A)** PCA plots of samples from each group(n=5-6). **(B)** Heatmap analysis of bile acid metabolism. **(C-H)** Abundance of cholic acid 7 sulfate (CA7S), Glycoursodeoxycholic Acid(GUDCA),Taurochenodesoxycholic Acid (TCDCA),Chenodeoxycholic Acid(CDCA),Taurocholic Acid(TCA),Ursodeoxycholic Acid(UDCA)(n=5-6). These results were presented as mean ± SD. *p < 0.05, **p < 0.01,***p < 0.001, compared with the CON group. #*p* < 0.05, ##*p* < 0.01, ###*p* < 0.001, compared with the LPS group.

We next employed targeted bile acid metabolism to investigate changes in bile acid profiles in feces, ultimately identifying 53 distinct bile acids. The PCA plot revealed marked differences across the three groups of metabolites (**Figure 5A**), with significant differences between the CON and LPS groups. Subsequently, heat maps(**Figure 5B**) and barplots (**Figures 5C–H**) were generated to illustrate the expression levels of all metabolites. The results revealed that, compared to the CON group, Cholic Acid 7 Sulfate (CA7S) was significantly decreased in the LPS but showed an increase in the MDD group. Notably, CA7S does not conform strictly to the conventional classifications of primary or secondary bile acids; instead, it is recognized as a metabolite or derivative of bile acid. Conversely, the abundance of Glycoursodeoxycholic Acid (GUDCA), Taurochenodesoxycholic Acid (TCDCA), Chenodeoxycholic Acid (CDCA), Taurocholic Acid (TCA) were significantly increased in LPS group and MDD decreased them. For Ursodeoxycholic Acid (UDCA), there were no change between LPS group and CON group, while MDD still decreased it. This suggests that MDD has a significant impact on both bile acids and bile acid metabolites. Overall, LPS significantly increased primary and secondary bile acids, whereas MDD markedly reduced their levels, which may be closely associated with alterations in gut microbiota.

### 3.7 MDD improved the gut microbiota and inhibited levels of bile acids

**Figure 6:**
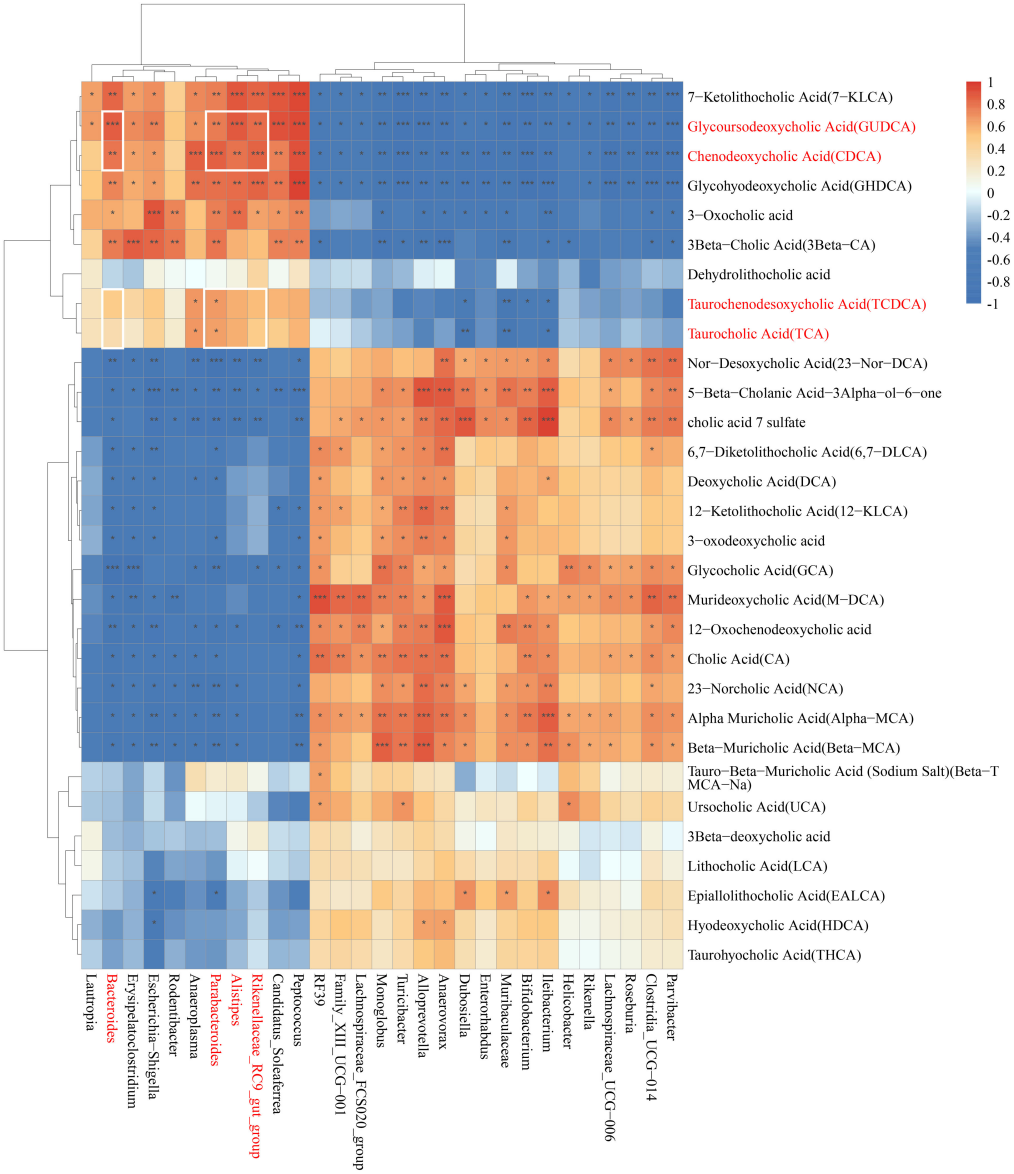
Correlation analysis between phylum and genus level of gut microbiota and bile acid metabolite. The correlation analysis between genus level of gut microbiota and bile acid metabolite.

Gut microbiota and bile acids are closely correlated, influencing each other’s generation and composition (Collins et al., 2023). *Bacteroides* and *Parabacteroides distasonis* alleviates obesity and metabolic dysfunctions via production of secondary bile acids (Xiang et al., 2022; Wei et al., 2023). The correlation analysis revealed that *Parabacteroides*, *Bacteroides, Alistipes*, and *Rikenellaceae_RC9_gut_group* exhibited a positive correlation with GUDCA, CDCA, TCDCA, and TCA (**Figure 6**).

### 3.8 MDD alleviated endothelial dysfunction by promoting the reduction of NETs

Based on our research on gut microbiota and bile acid metabolism, we found that MDD could improve gut microbiota structure and alter primary bile acids and their derivatives. However, the mechanisms remain unclear. Previous studies indicated that NETs may promote inflammation, activate endothelial cells, or directly damage the endothelial glycocalyx, impairing endothelial function and facilitating sepsis ultimately (Ma et al., 2019; Iba et al., 2024).

**Figure 7:**
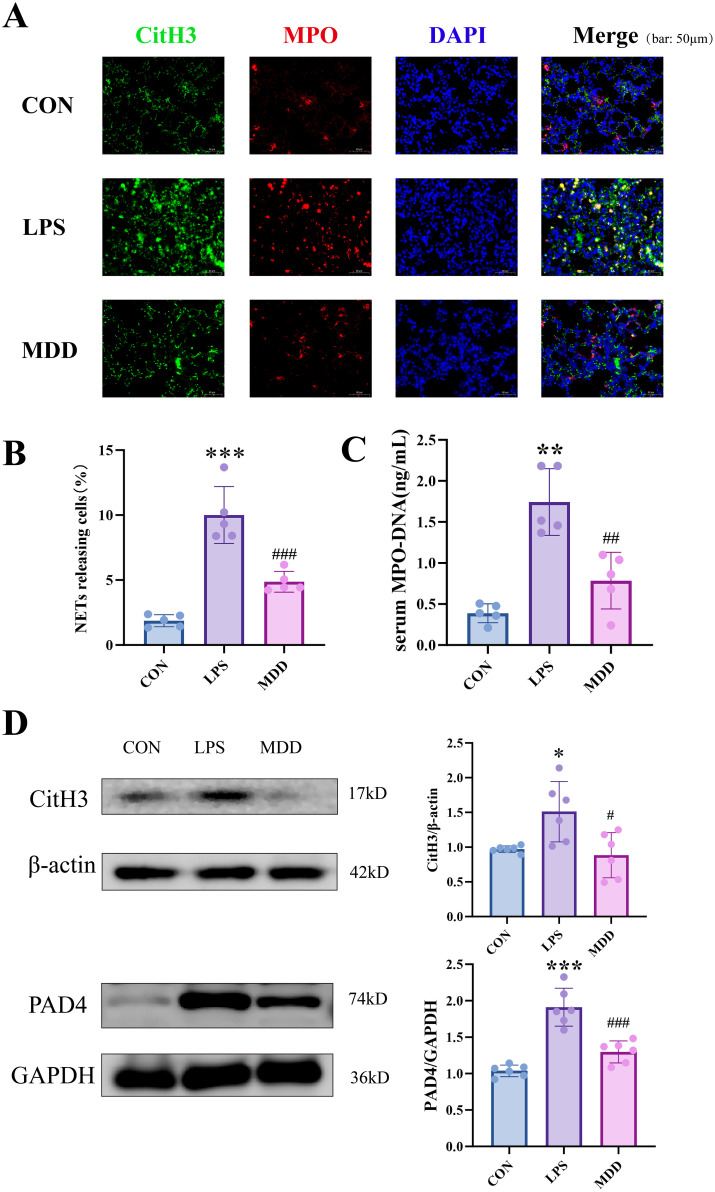
MDD alleviated endothelial dysfunction by promoting the reduction of NETs. **(A, B)** The co-expression of CitH3 and MPO in lung tissue(n=6,bar:50µm). **(C)** The MPO-DNA complex in serum. **(D)** The relative protein expression of CitH3 and PAD4 in lung tissue(n=5). These results were presented as mean ± SD. *p < 0.05, **p < 0.01,***p < 0.001, compared with the CON group. #*p* < 0.05, ##*p* < 0.01, ###*p* < 0.001, compared with the LPS group.

It is reported that NETs is a significant mechanism contributing to endothelial dysfunction (Papayannopoulos, 2018). The citrullinated histone H3 (CitH3) serves as a biomarker for the formation of NETs (Masuda et al., 2016). Immunofluorescence experiments demonstrated a predominant growth in the co-expression of CitH3 and the MPO in the LPS, which was reversed by MDD (**Figures 7A, B**). Myeloperoxidase (MPO) is an enzyme predominantly localized in neutrophils and monocytes. The MPO-DNA complex, which signifies the co-localization of neutrophil-derived proteins and extracellular DNA, is an indicator of NETs formation (Masuda et al., 2016). Accordingly, we measured the concentration of the MPO-DNA complex in mouse serum and found a remarkable increase in the LPS group, which could be reduced by MDD (**Figure 7C**). Western blot analysis also shows MDD decrease the CitH3 of lung. (**Figure 7D**). It has been reported that peptidyl arginine deiminase 4 (PAD4) citrullinates histones, leading to the loosening of chromatin structure and promoting DNA release, which is a key step in the formation of NETs (Liu et al., 2024). By analyzing its expression in lung tissue, we observed results consistent with those of CitH3. LPS significantly increased its expression, whereas MDD treatment effectively reversed this effect. These findings suggest that MDD may reduce SI-ALI by mitigating excessive NETs to improve endothelial dysfunction.

### 3.9 MDD primarily acted on the FXR/TLR4/MYD88 signaling pathway

**Figure 8:**
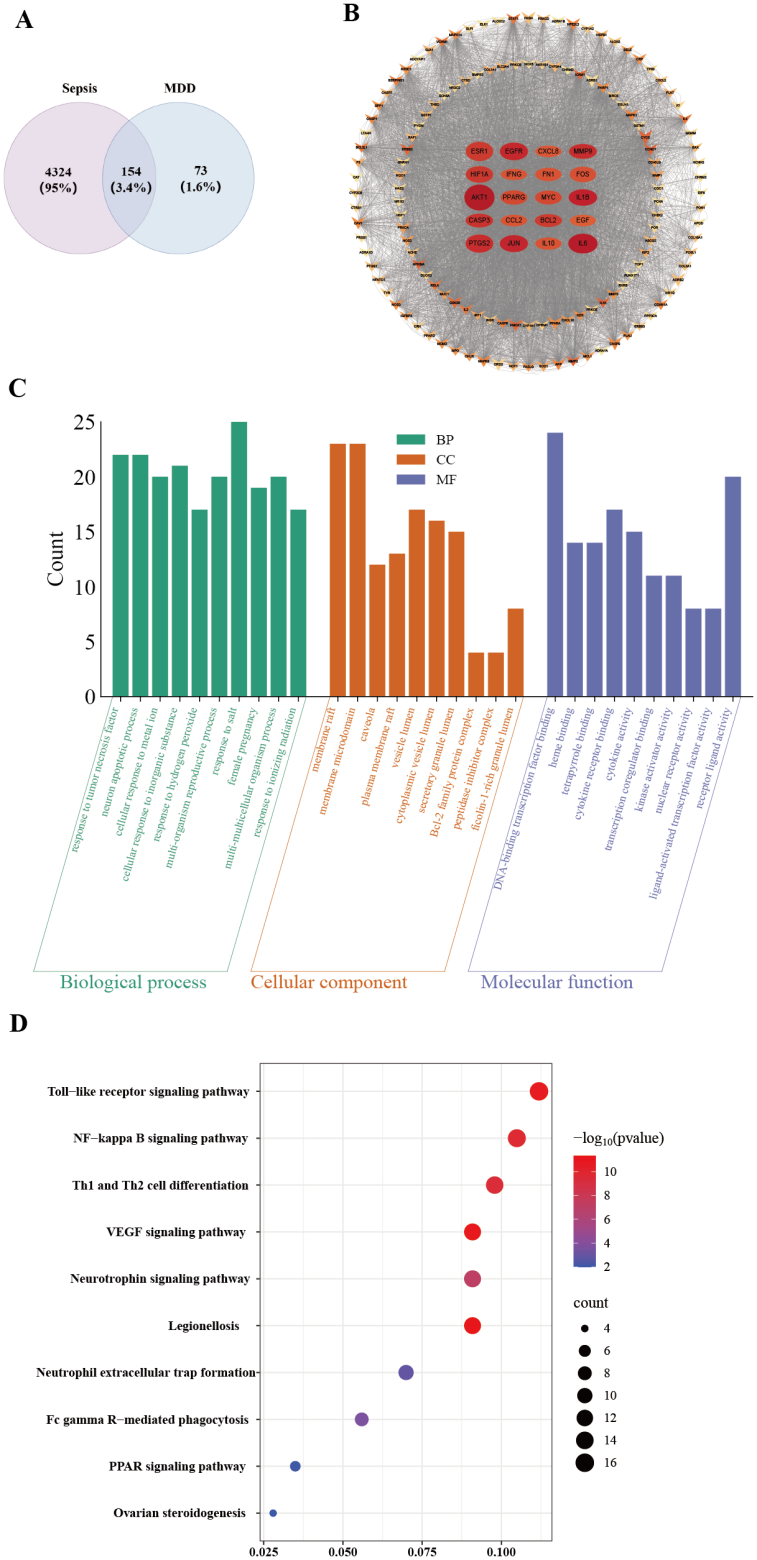
Network pharmacological analysis of MDD and sepsis. **(A)** Intersection targets of sepsis and MDD; **(B)** PPI network diagram of intersection targets of sepsis and MDD; **(C)** Bar charts of biological processes, cellular components, and molecular functions from GO enrichment analysis; **(D)** Bubble chart of the top 10 KEGG enrichment analyses.

We obtained 4,478 sepsis-related gene targets and 227 MDD-associated target genes through database searching. Using Jvenn online software analysis, we identified 154 overlapping genes between the core drug target genes and sepsis targets (**Figure 8A**). Subsequently, these 154 overlapping genes were imported into the STRING database to construct a PPI network (**Figure 8B**). The results of GO functional analysis revealed that these genes or proteins were primarily involved in biological processes such as tumor necrosis factor response, neuronal apoptosis, and metal ion response. They were localized in cellular components, including membrane rafts, cytoplasmic vesicles, and granular lumina, and possess molecular functions like DNA-binding transcription factor binding, cytokine activity, and kinase activator binding, suggesting their critical roles in signal transduction, immune regulation, and gene expression control (**Figure 8C**). Moreover, KEGG pathway enrichment analysis indicated that the core targets were significantly enriched in the Toll-like receptor signaling pathway, further untangling their potential regulatory mechanisms in immune and inflammatory responses (**Figure 8D**). The key upregulated genes in the TLR signaling pathway were visualized using the multiprotein Profiler 1.0 (MPP) web server (https://mproteinprofiler.microbiologyandimmunology.dal.ca/)(Sganzerla Martinez et al., 2024), and their protein physicochemical characteristics were displayed, as shown in **Supplementary file 9-10**.

**Figure 9:**
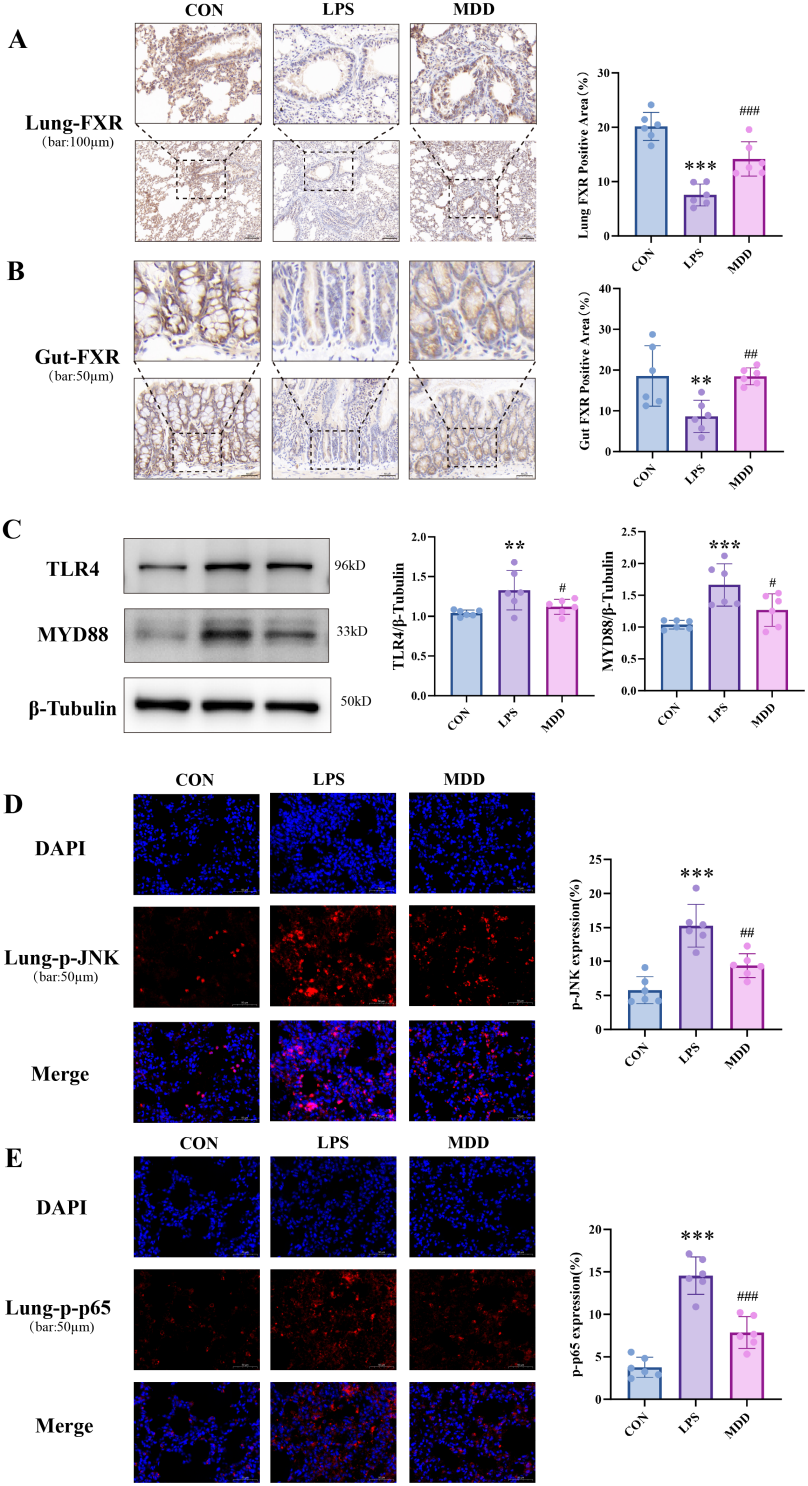
MDD primarily acted on the FXR/TLR4/MYD88 signaling pathway. **(A)** FXR expression in lung tissue(n=6,bar:100µm); **(B)** FXR expression in gut(n=6,bar:50µm); **(C)** Expression levels of TLR4/MYD88 in lung tissue(n=6). **(D)**The p-JNK and **(E)** p-p65 expression in lung tissue(n=6,bar:50µm)These results were presented as mean ± SD. *p < 0.05, **p < 0.01,***p < 0.001, compared with the CON group. #*p* < 0.05, ##*p* < 0.01, ###*p* < 0.001, compared with the LPS group.

FXR serves as a crucial sensor for bile acids and can be inhibited by *Parabacteroides distasonis* (Zhao et al., 2023). According to research findings, mice overexpressing FXR exhibit enhanced tolerance to sepsis (Hao et al., 2017; Li et al., 2023). In this study, we examined the expression of FXR in intestinal and lung tissues. The results indicated a significant reduction in both the lungs and intestines of the LPS group compared to the CON group (**Figures 9A, B**). Conversely, MDD treatment increased FXR expression, primarily in lung epithelial cells (**Figure 9B**). Furthermore, the toll-like receptor signaling pathway identified through enrichment analysis in network pharmacology has been experimentally validated. Toll-like receptor 4(TLR4) is a crucial member of the toll-like receptor family, while myeloid differentiation primary response 88(MYD88) serves as a key adaptor protein downstream. The TLR4/MYD88 signaling pathway has been identified as a critical regulator of NETs in sepsis. Studies have demonstrated that MYD88-deficient mice exhibit reduced formation of lethal intravascular NETs, consequently attenuating septic progression, which underscores the pivotal role of MYD88 in NET hyperactivation (Hu and Mao, 2016).Our results indicated a significant upregulation of TLR4 and MYD88 expression in the LPS group, whereas a downregulation was observed in the MDD group (**Figure 9C**). Furthermore, the TLR4 signaling pathway is closely associated with the expression of other inflammatory mediators, including NF-κB and IL-1β, which also participate in the formation and modulation of NETs (Englert et al., 2025). The immunofluorescence detection of phosphorylated p65 (p-P65) and phosphorylated JNK (p-JNK) in lung tissues was performed. The results indicated that LPS significantly increased the positive signals of p-P65 and p-JNK in lung tissues, whereas MDD treatment markedly attenuated the expression of these phosphorylation markers (**Figure 9D, E**). Therefore, the FXR/TLR4/MYD88 signaling pathway may serve as a crucial mechanism through which MDD modulates SI-ALI, and this mechanism is closely associated with *Parabacteroides distasonis*.

## 4 Discussion

**Figure 10:**
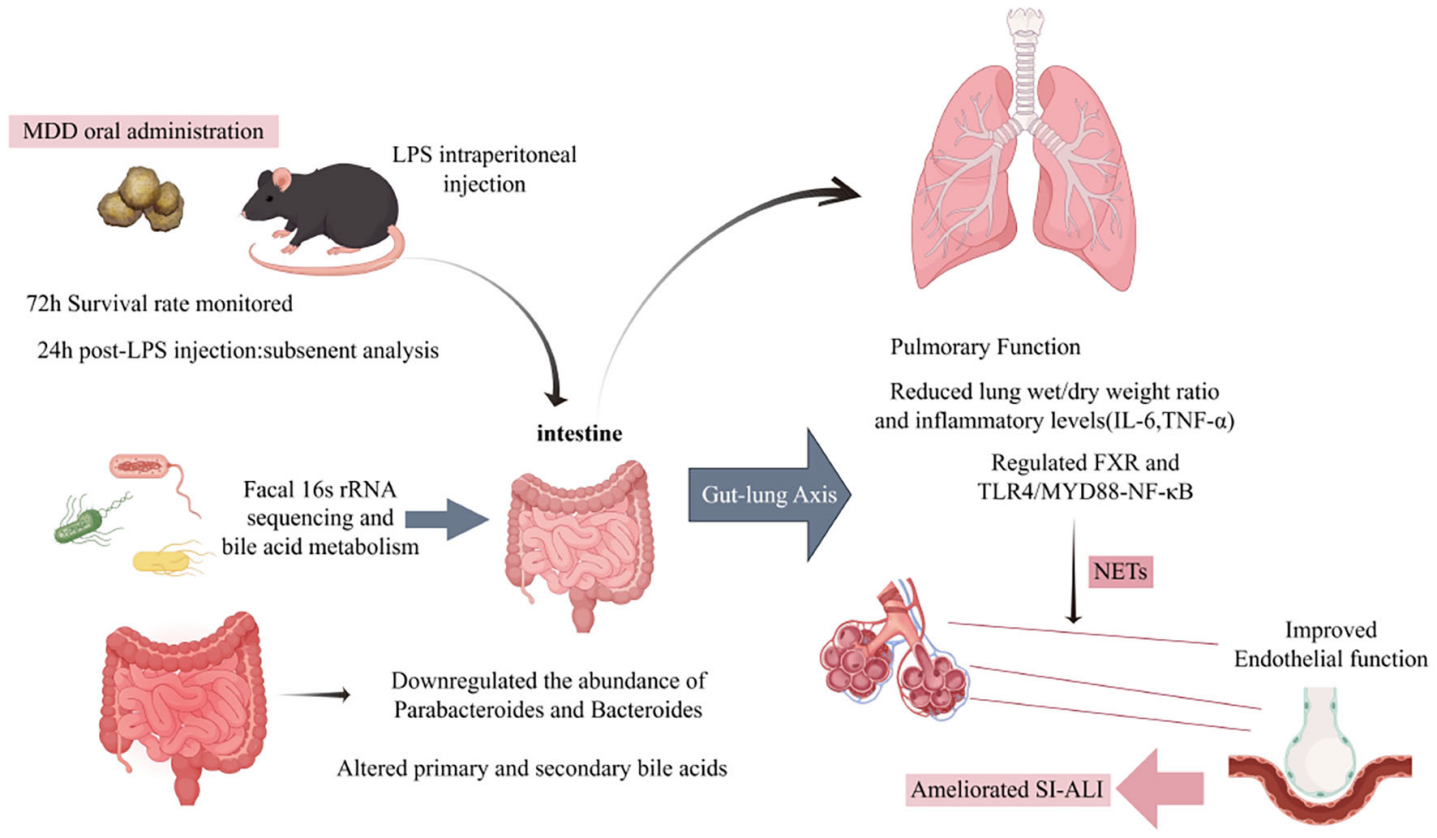
The potential mechanism of MDD in alleviating SI-ALI partially through influencing bile acids metabolism and gut microbiota.

**Figure 11:**
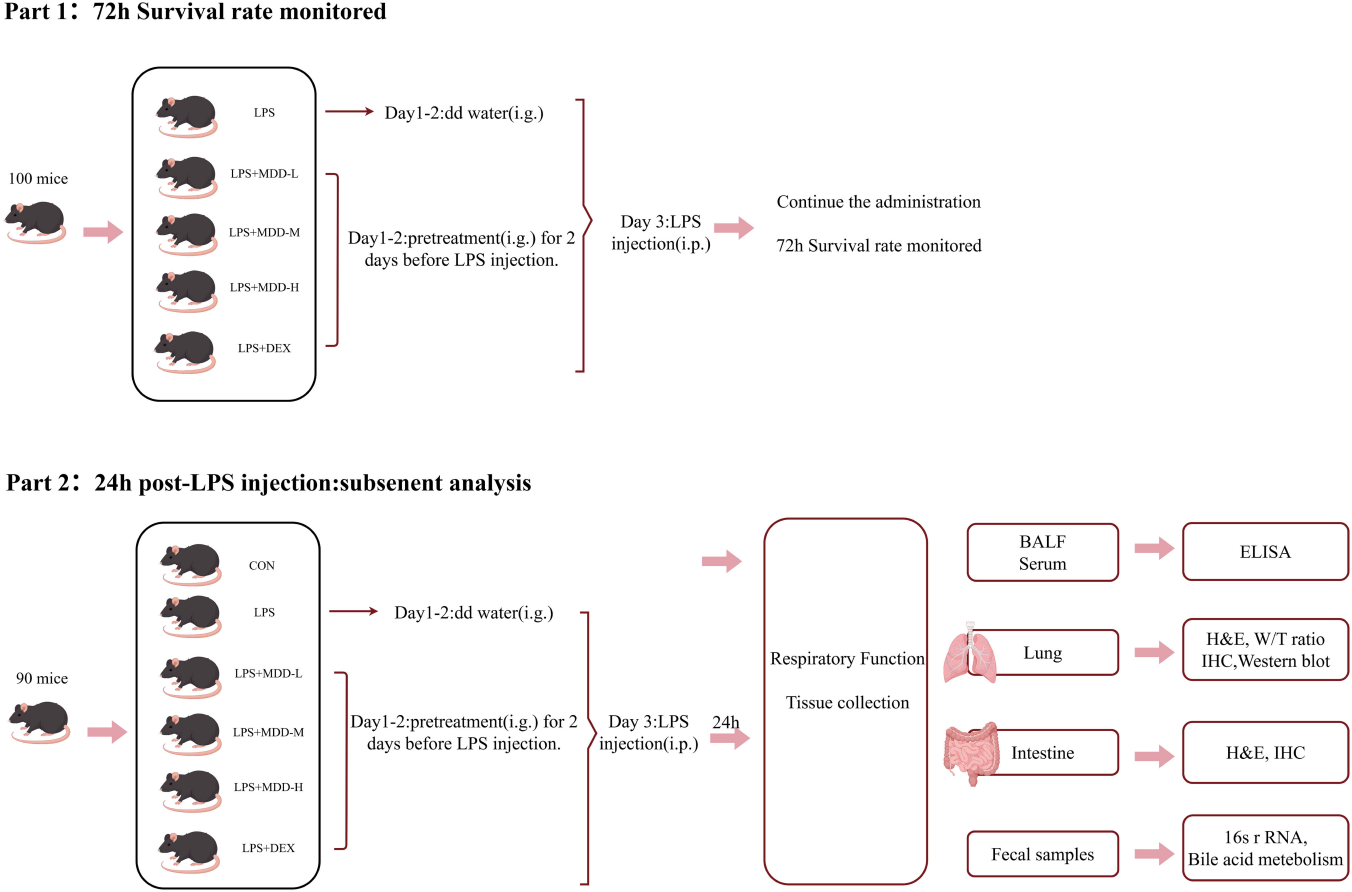
Methodology of this study.

In this study, we employed a comprehensive approach combining 16S rRNA sequencing and targeted bile acid metabolomics to investigate the gut-lung axis correlation and the therapeutic role of anti-SI-ALI. Our mechanistic findings demonstrated that MDD exerts protective effects against LPS-induced SI-ALI through multiple pathways. Specifically, MDD downregulates NETs activation primarily via modulation of the FXR/TLR4/MYD88 signaling pathway (**Figures 10**, **11**). Furthermore, our results show that MDD significantly decreases fecal levels of primary and secondary bile acids and modulates gut microbiota composition in SI-ALI mice, particularly by reducing the abundance of *Parabacteroides* and *Bacteroides* genus. These combined effects contribute to SI-ALI alleviation through two primary mechanisms: mitigation of intrapulmonary NETs formation and reduction of pulmonary endothelial dysfunction. Importantly, our findings establish that the therapeutic efficacy of MDD in SI-ALI is significantly associated with its dual regulatory effects on gut microbiota composition and bile acid metabolism.

In this study, MDD significantly improved the 72-hour survival rate, indicating its comprehensive protective effect against acute inflammatory outburst and its downstream complications. The increase in survival was consistent with reductions in inflammatory factors, decreased NETs, and recovery of pulmonary function, suggesting that MDD alleviates SI-ALI not through a single pathway but via multi-target synergistic mechanisms. Improvements in respiratory parameters such as tidal volume and peak inspiratory flow reflected the restoration of gas exchange and lung compliance, which likely resulted from reduced pulmonary interstitial edema and inflammatory cell infiltration, thereby lowering alveolar–capillary barrier permeability. Decreases in the W/D weight ratio and BALF protein further demonstrated the alleviation of microvascular permeability and pulmonary edema, supporting its protective effect on the microvascular barrier. Meanwhile, downregulation of TNF-α and IL-6 indicated suppression of the early inflammatory amplification loop. This anti-inflammatory effect corresponded with improvements in endothelial markers—MDD reversed the LPS-induced upregulation of VCAM-1 and downregulation of VE-cadherin, demonstrating that it not only reduces pro-inflammatory stimuli but also preserves cell–cell adhesion and endothelial integrity. To explore the upstream factors underlying these outcomes, the following section will focus on alterations in gut microbiota and bile acid metabolism, as well as evidence supporting their roles as potential mediating mechanisms. A recent study has identified that phenylpyruvate, secreted by intestinal *Candida albicans*, may target SIRT2 in the context of sepsis (Gu et al., 2023). Moreover, the production of hyodeoxycholic acid (HDCA) functions as an endogenous inhibitor of TLR4, potentially mitigating systemic inflammation associated with sepsis (Li J. et al., 2023). A recent study revealed that rhamnose, a bacterial carbohydrate metabolite, could enhance the phagocytic capacity of macrophages by directly binding to and activating SLC12A4 within these cells, which protect the host against sepsis (Li D. et al., 2024). The above findings overlap in their assertion of the significant role of gut microbiota and their associated metabolites in the pathogenesis of sepsis. Nonetheless, the application of fecal microbiota transplantation(FMT) in patients presents considerable challenges (Biemond et al., 2023), emphasizing the need to investigate straightforward and practical approaches for modulating gut microbiota within the framework of sepsis treatment. To address this challenge, the present study employed a TCM formulation to evaluate its effects on gut microbiota, aiming to untangle the mechanism underlying lung-gut co-therapy and develop an alternative rapid strategy for FMT.

Previous studies have demonstrated that MDD exhibits promising efficacy in clinical disease treatment (Chen et al., 2016, 2018). However, its specific clinical mechanisms remain unclear, and direct evidence for the role of traditional Chinese medicine in alleviating pulmonary and intestinal complications associated with sepsis is relatively limited. In this study, we found that MDD could improve *Parabacteroides* and *Bacteroides* on genus level. Studies have revealed that *Parabacteroides* exhibits a dual role. Lower abundance is showed in patients with obesity, inflammatory bowel disease (IBD), and metabolic syndrome, whereas it is increased in individuals with psoriasis, neonatal cholestasis, alopecia areata, hypertension, and polycystic ovary syndrome (PCOS) (Ezeji et al., 2021). This suggests that *Parabacteroides* may display both probiotic and pathogenic properties depending on virous host. Notably, it promotes the synthesis of secondary bile acids (Wang et al., 2019), which aligns with the findings of this study.

Similarly, *Bacteroides*, a prominent genus within the gut microbiota, functions as an opportunistic pathogen like *Parabacteroides* (Zafar and Saier, 2021). It is now understood that *Bacteroides* play a crucial role in maintaining gut health and facilitating digestion in healthy individuals. However, under compromised immune function, broad-spectrum antibiotic employment, or other adverse conditions, *Bacteroides* may overgrow, increasing the risk of sepsis (van der Poll et al., 2021; Mu et al., 2022). Such over-proliferation can lead to bacterial translocation and toxin release, triggering a systemic inflammatory response that may ultimately result in multi-organ dysfunction, posing a serious threat to patient survival. In this respect, a high abundance of *Bacteroides* has been associated with increased likelihood of achieving a Sequential Organ Failure Assessment (SOFA) score of 10 or more during hospitalization, a higher proportion of patients with ICU stays exceeding 30 days, and elevated mortality rates (Sun et al., 2023). Given that our results indicated that MDD could reduce the abundance of *Bacteroides* in mice, we hypothesize that the alleviation of SI-ALI by MDD may be associated with *Parabacteroides* and *Bacteroides*.

The role of bile acids in lung injury exhibits a double-edged effect. On one hand, certain bile acids demonstrate significant therapeutic potential. For instance, GCDCA significantly suppress NLRP3 inflammasome activation by activating the TGR5 receptor pathway (Zhang Z. Y. et al., 2024). On the other hand, bile acids can induce lung injury through various mechanisms, including interaction with the secretory phospholipase A2 (sPLA2) pathway, disruption of alveolar surfactant function, modulation of inflammatory responses and local immunity, as well as direct cytotoxic effects (De Luca et al., 2022).A high concentrations-bile acids exhibit cytotoxicity, Deoxycholic acid (DCA) and CDCA were found to induce robust secretion of pro-inflammatory cytokines IL-1α and IL-1β from bone marrow-derived dendritic cells (Oleszycka et al., 2023). Our findings demonstrate that MDD reduces the concentrations of both primary and secondary bile acids, particularly the pathogenic bile acid CDCA. This reduction in concentration markedly attenuates the cytotoxic effects of bile acids on cells.

Significant alterations in gut microbiota composition were observed in the SI-ALI, characterized by a marked increase in the abundance of opportunistic pathogens such as *Parabacteroides* and *Bacteroides*. These microbiota harbor abundant BSH and 7α-dehydroxylase, which accelerate the conversion of primary bile acids to secondary bile acids. Notably, at physiological concentrations, bile acids exert anti-inflammatory and metabolic regulatory roles by activating receptors such as FXR and TGR5. However, elevated bile acid levels can trigger the explosive release of pro-inflammatory cytokines IL-1β and TNF-α, directly damaging tissue barriers. This study demonstrated that increased secondary bile acids in the intestinal tract of the LPS group were positively correlated with pulmonary inflammatory infiltration and endothelial injury, suggesting that microbiota-mediated excessive bile acid accumulation may be a key driver of SI-ALI.

Notably, it was observed that the protective effect of MDD against lung injury was not limited to intestinal microenvironment regulation. Further investigations revealed that MDD significantly suppressed the formation of NETs in the lungs-a key effector mediating endothelial injury and pulmonary microcirculatory dysfunction. The NETs markers CitH3 and MPO-DNA complexes were markedly elevated in the LPS group but were substantially inhibited following MDD intervention. This change was probably associated with MDD-mediated reduction in *Parabacteroides*, activation of FXR expression in both lung and intestinal tissues, and suppression of the TLR4/MYD88 signaling pathway. Previous studies have demonstrated that FXR, functioning as a bile acid nuclear receptor, can attenuate excessive inflammatory responses by antagonizing the TLR4 pathway (Xiao et al., 2025). Importantly, TLR4/MYD88 activation has been identified as the core driver of NETs formation (Suprewicz et al., 2025).

NETs, released by neutrophils, are web-like structures primarily composed of DNA, histones, and granular proteins. They play a pivotal role in defending against infections by physically entrapping and chemically killing pathogens (Brinkmann et al., 2004). However, excessive formation or clearance impairment of NETs can lead to tissue damage and inflammatory responses, contributing to pathological processes such as autoimmune diseases, thrombosis, and cancer progression (Papayannopoulos, 2018). Research indicated that NETs play a vital part in developing sepsis and the associated endothelial dysfunction (Zhang et al., 2023). Although a moderate level of NETs is conducive to immune function, excessive and prolonged NETs release can amplify inflammatory processes (Doring et al., 2020; Yang et al., 2020). The primary components of NETs, including extracellular DNA, histones, neutrophil elastase (NE), and MPO, can directly damage pulmonary microvascular endothelium, increase vascular permeability, and induce cell death (Zhou et al., 2024). Concurrently, they upregulate adhesion molecules such as VCAM-1 (Folco et al., 2018; Zhang et al., 2023), recruit inflammatory cells, and provide a scaffold for platelets and coagulation factors, thereby promoting immunothrombosis (Middleton et al., 2020).This cascade leads to impaired microcirculatory perfusion and tissue hypoxia. Clinical and experimental data indicate that NETs levels are positively correlated with disease severity and prognosis in severe lung injuries such as ARDS and COVID-19 (Middleton et al., 2020; Cesta et al., 2023).Inhibition of NETs formation or acceleration of their clearance has been shown to mitigate endothelial injury and improve lung function. NETs contribute to endothelial dysfunction, further worsening sepsis-induced lung injury.

FXR, a nuclear receptor belonging to the nuclear receptor superfamily, is primarily expressed in the liver, intestine, and kidney, although studies have confirmed its expression in lung tissue as well (Li Z. et al., 2023). This process can be inhibited by the highly abundant *Parabacteroides* (Zhao et al., 2023), which may represent the core mechanism through which MDD modulates FXR signaling. As a critical regulatory factor in bile acid metabolism, FXR plays a significant role in inflammation and metabolic regulation. Research indicates that mice overexpressing FXR demonstrate significant resistance to endotoxemia (Hao et al., 2017), suggesting a potential role of FXR in inhibiting systemic inflammatory responses. TLR4, a pattern recognition receptor, primarily recognizes LPS, a component of the outer membrane of Gram-negative bacteria. TLR4 binds to the adaptor protein MYD88 through its Toll/IL-1 Receptor homologous domain, initiating downstream signaling pathways and inducing the expression of proinflammatory cytokines such as TNF-α and IL-6 (Kawai and Akira, 2011). Overactivation of TLR4 is closely associated with a high incidence of sepsis (Kondo et al., 2012). Studies have shown that FXR can effectively inhibit inflammatory responses by suppressing the TLR4/MYD88 signaling pathway (Xiao et al., 2025). Furthermore, previous research has confirmed that the TLR4-dependent pathway can significantly enhance neutrophil migration and promote the formation of NETs (Suprewicz et al., 2025).This suggests that the FXR/TLR4/MYD88 pathway serves as a critical axis through which MDD modulates NETs and their downstream responses.

Based on the above findings, this study proposes that MDD alleviates SI-ALI through a dual synergistic mechanism: first, by directly reshaping the gut microbiota to reduce the accumulation of pro-inflammatory and cytotoxic bile acids, thereby lowering the systemic inflammatory burden at its source; second, by indirectly restoring FXR activity, which suppresses the TLR4/MYD88 signaling pathway, reduces PAD4-mediated NETs release, and consequently protects pulmonary microvascular endothelium, decreases permeability, and improves lung function. The composition of MDD is diverse. We detected the Anthraquinones, anthrones, Flavones and fatty acids, which are closely related to sepsis and ALI. Studies have shown that the delivery of baicalin in a sepsis mouse model via nanomaterials could significantly promote the polarization of macrophages from the proinflammatory M1 phenotype to the anti-inflammatory M2 phenotype, thereby effectively inhibiting inflammatory responses and exerting a therapeutic effect (Zhou M. et al., 2025). Emodin was also found to significantly alleviate LPS-induced ALI and exert a protective effect by regulating the JNK/Nur77/c-Jun signaling pathway (Xie et al., 2022). Besides, it was reported that emodin regulate the level of severe acute pancreatitis-related exosomes and their proinflammatory components, inhibit the proinflammatory polarization of alveolar macrophages, and thereby alleviate ALI (Hu et al., 2022).MDD may exhibit its unique therapeutic advantages based on multi-component synergistic interactions by regulating the gut-lung axis function through multiple targets and intervening in the pathological process of SI-ALI.

However, this study has certain limitations that should be acknowledged. Firstly, MDD, being a multi-component traditional Chinese medicine, comprises a complex mixture of bioactive compounds. Although some components have been identified through UPLC-MS analysis, the interactions among these components and their specific contributions to the overall therapeutic effect remain incompletely understood, requiring further investigation. Secondly, this study represents a preliminary exploration primarily aimed at untangling the potential mechanisms through which MDD alleviates SI-ALI by modulating gut microbiota and bile acid metabolism. Therefore, the findings of this study need to be further validated through larger-scale clinical studies and more in-depth experimental verification to confirm its efficacy and mechanisms. It should be noted that 16S rRNA sequencing is inherently a relative quantification method, which may introduce limitations in interpreting changes in community abundance. Subsequent studies could further employ qPCR to perform targeted quantification of key genera and species to strengthen the conclusions. Besides, the MDD-M group exhibited the optimal therapeutic efficacy, while the high- and low-dose groups showed relatively weaker effects. This observation suggests that the action of MDD may not follow a strictly dose-dependent pattern. Similar phenomena are not uncommon in multi-component TCM formulations (Lin et al., 2025; Liu et al., 2025), which may be attributed to their multi-target, multi-pathway regulatory characteristics and saturation effects *in vivo* metabolism. On the one hand, certain components at higher doses may lead to competition among pharmacological pathways or increased metabolic burden, thereby attenuating the overall synergistic effect. On the other hand, insufficient exposure to active constituents at lower doses may also limit efficacy. From the perspective of TCM theory, a high dose of MDD may exert overly strong purgative effects, potentially impairing the body’s healthy qi (zhengqi), whereas a low dose may provide insufficient purgative action to effectively eliminate the pathogenic factors. In summary, while this study primarily focused on the therapeutic effects of MDD, it did not systematically evaluate the safety and potential side effects of high doses, which represents an important direction for future research. More detailed histopathological examinations and monitoring of biochemical indicators such as liver function in the high-dose group will help clarify its safety window.

## 5 Conclusions

The study demonstrates that MDD can significantly alleviate SI-ALI, as evidenced by improved histopathology, reduced inflammatory cytokines, and attenuated pulmonary edema. The remodeling of the intestinal microbiota and alterations in targeted bile acids, combined with molecular biology validation, suggest that MDD may modulate the gut microbiota–bile acid axis, activate FXR, and inhibit the TLR4/MYD88 inflammatory pathway, thereby reducing PAD4-mediated NETs formation and ultimately ameliorating endothelial dysfunction.

However, this study has several limitations. First, the interactions among the components of MDD and their respective contributions to therapeutic efficacy remain unclear, necessitating further research on the interactions between individual compounds and the complete formula. Second, more rigorous mechanistic validation is required regarding dose-response relationships, safety assessments, and the translation of findings from animal models to clinical applications. Third, the limitations of the relative quantification method using 16S rRNA sequencing suggest that key bacterial genera or species should be further validated using targeted approaches such as quantitative polymerase chain reaction (qPCR) or metagenomics.

## Data Availability

The original contributions presented in the study are included in the article/**Supplementary Material**. Further inquiries can be directed to the corresponding authors.
