# Supplementary material for: Modified Dachengqi Decoction ameliorates sepsis-induced lung injury via the gut microbiota-bile acid axis
Source: Front Cell Infect Microbiol. 2026 Feb 6;16:1661639. doi: 10.3389/fcimb.2026.1661639 (PMC12920581; doi:10.3389/fcimb.2026.1661639)
Supplement: Supplementary file 5 [file DataSheet5.docx]

Graphical representation of MPP output for
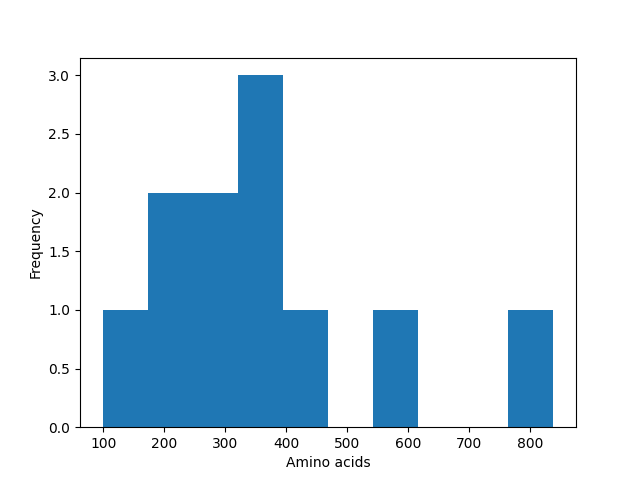
key up-regulated genes.

(a)Lenth of amino acids
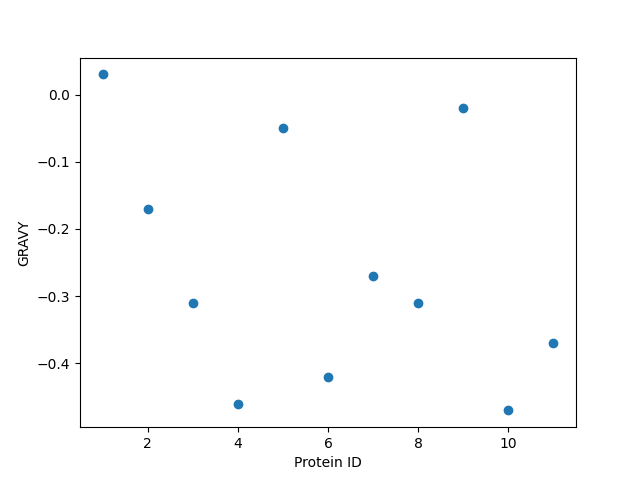


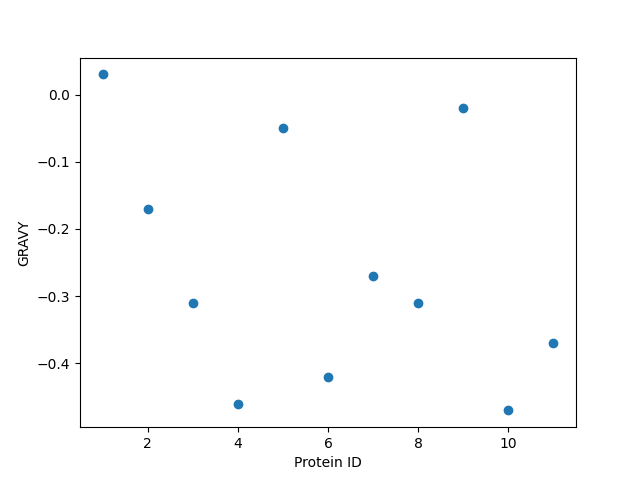


(b)GRAVY


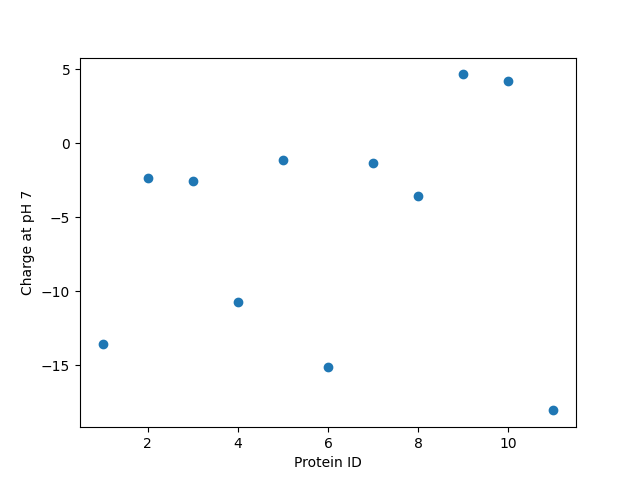


(c)Charge at pH 7


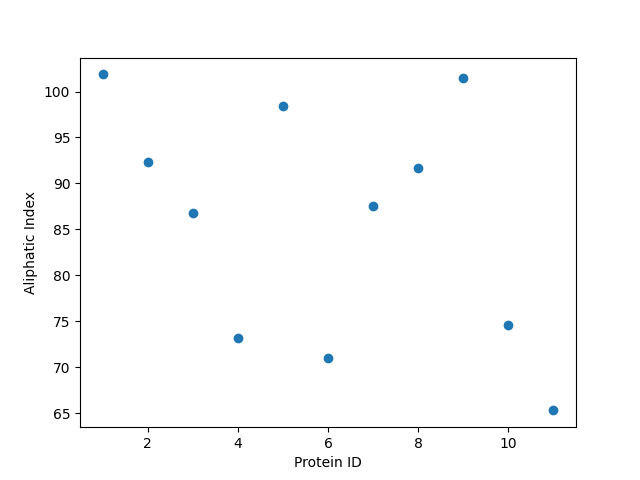


(d)Aliphatic index


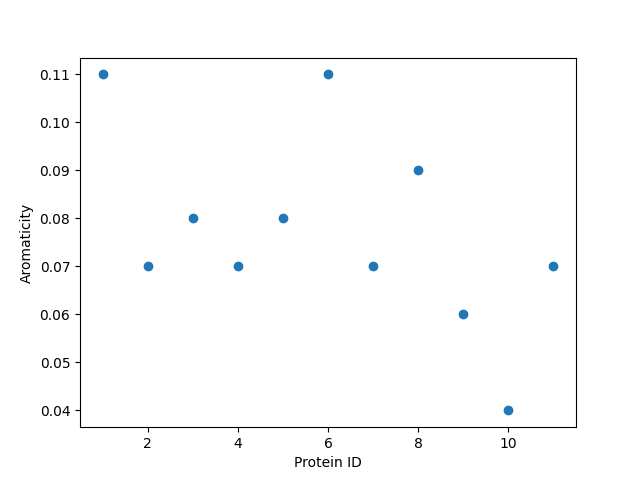


(e) Aromaticity


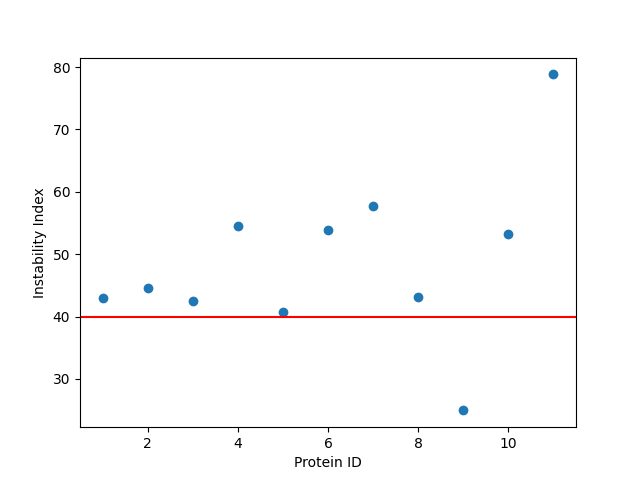


(f)Instability index


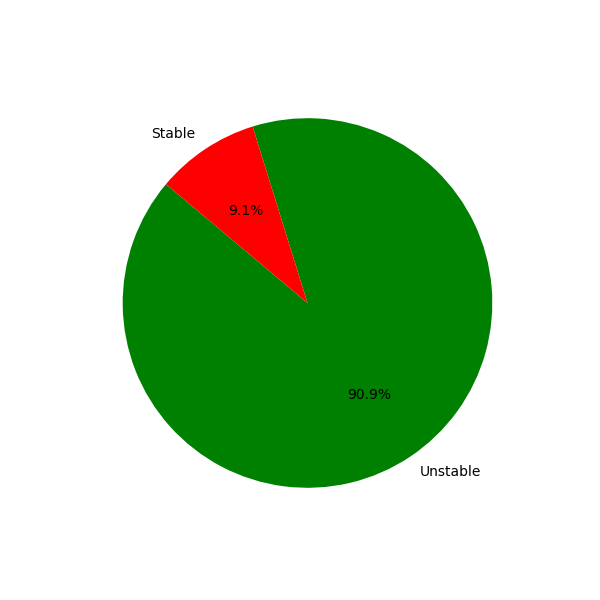


(g)Stability


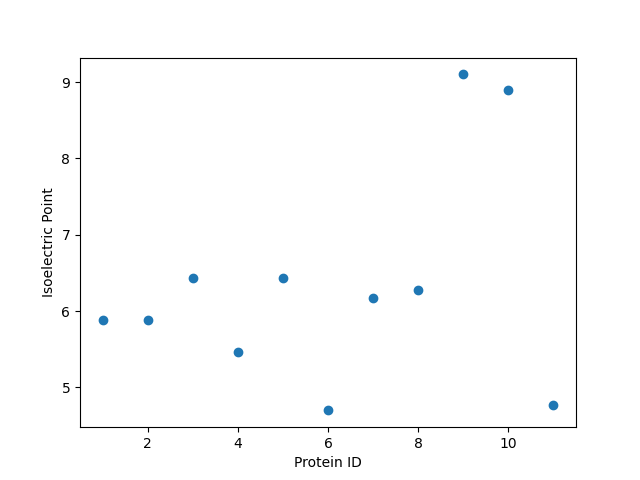


(h)Isoelectric point


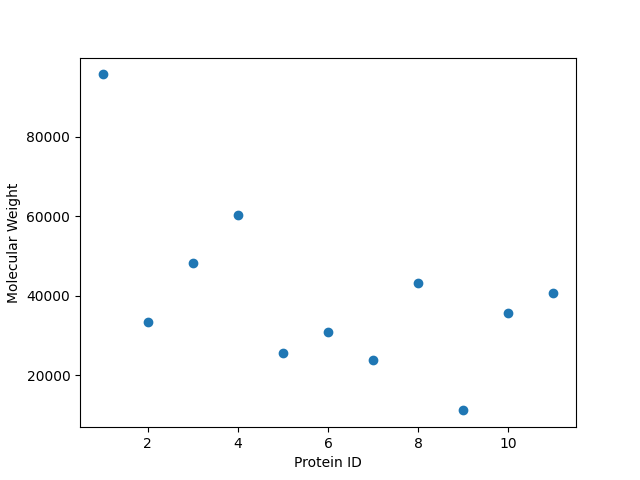


(i)Molecular weight
